# Milling Mechanism and Chattering Stability of Nickel-Based Superalloy Inconel 718

**DOI:** 10.3390/ma16175748

**Published:** 2023-08-22

**Authors:** Jin Zheng, Yaoman Zhang, Hanying Qiao

**Affiliations:** School of Mechanical Engineering & Automation, Northeastern University, Shenyang 110819, China; 2270293@stu.neu.edu.cn (J.Z.); 2070194@stu.neu.edu.cn (H.Q.)

**Keywords:** nickel-based superalloy, finite element analysis, chattering analysis, milling stability, parameters optimization

## Abstract

Nickel-based superalloy Inconel 718 is widely used in the aerospace industry for its excellent high-temperature strength and thermal stability. However, milling Inconel 718 presents challenges because of the significantly increased cutting force and vibration, since Inconel 718 is a typical difficult-to-machine material. This paper takes the milling process of Inconel 718 as the research object, initially, and a milling force model of Inconel 718 is established. Subsequently, the finite element analysis method is used to analyze the stress field, temperature field, and milling force in the milling process of Inconel 718. Building upon this, a dynamic equation of the milling of Inconel 718 is established, and based on the modal experiment, stability lobe diagrams are drawn. Furthermore, milling experiments on Inconel 718 are designed, and the results calculated using the milling force model and finite element analysis are verified through comparison to the experimental results; then, the fmincon optimization algorithm is used to optimize the processing parameters of Inconel 718. Eventually, the results of the multi-objective optimization illustrate that the best processing parameters are a spindle speed of 3199.2 rpm and a feed speed of 80 mm/min with an axial depth of cut of 0.25 mm. Based on this, the best machining parameters are determined, which point towards an improvement of the machining efficiency and quality of Inconel 718.

## 1. Introduction

Nickel-based superalloy Inconel 718 is widely used in the aerospace industry because of its extraordinary high-temperature strength, thermal stability, heat fatigue resistance, corrosion resistance, creep resistance, and oxidation resistance, especially in the manufacturing of key components for the aerospace industry, such as turbine blades, combustion chamber components, and gas turbines [1,2,3,4,5,6]. Common processing methods for Inconel 718 are mainly casting [7,8], forging [9], additive manufacturing [10,11], and electrical discharge machining [12]. However, each of these machining methods suffers from flaws, like low dimensional accuracy; inability to process complex geometry; high machining costs; and containing heat-affected areas, slow machining speed, and poor surface quality, respectively. Although utilizing the milling method can effectively avoid the abovementioned problems, in the cutting process Inconel 718 is prone to serious tool wear [13], high thermal stress, high cutting forces [14], vibration, and other phenomena [15]. To address these challenges and improve the machinability of Inconel 718, the milling mechanisms and chattering stability of Inconel 718 need to be investigated. Based on this, the optimized processing parameters can be determined by analyzing the influence of different processing parameters, such as spindle speed, feed per tooth, radial depth of cut, and axial depth of cut on the cutting force, temperature, stress, and deformation, including machining quality during manufacturing.

Accurate modeling of the milling force can help adjust the cutting parameters to ensure high-quality machining results; therefore, it is especially necessary to establish an accurate milling force model. At present, the milling force models proposed by scholars mainly include empirical models, physical analytical models, and finite element models.

The empirical model of milling force is a model derived by fitting milling test data and is mainly used to predict the magnitude of milling force. Ding [16] and Lei [17] used orthogonal tests to obtain the milling force coefficients and established an empirical model of milling force. Bergmann [18] developed a nonlinear empirical model of milling force and improved the prediction of stability limits achieved by parameterizing a milling force model for stability analysis. Zhao [19] established an empirical model of milling force for ultrasonic vibration milling of titanium alloy and explored the effects of different machining parameters on milling force. Liang [20] proposed an empirical model of cutting forces in micro-end-milling operations that takes into account the parameters of feed per tooth per tool radius, the true trochoidal nature of the tool edge path, and the chip thickness. In fact, the empirical model of milling force relies on a large amount of experimental data and has obvious limitations, which cannot reveal the dynamic characteristics and mechanism of cutting and can only be used to calculate the average milling force, rather than accurately predict the instantaneous cutting force during the milling process.

A physical analytical model of milling force is a model that describes the cutting forces generated during the milling process with the help of mathematical analytical methods. The physical analytical model of milling force is usually based on the basic theories of machining principles and material mechanics. Jiang [21] proposed that the projected area of the milling shear surface in three directions determines the milling force in three directions, and established an analytical model of milling force based on the shear surface. Madajewski [22] presented a computational method based on a combination of finite element analysis and classical analytical methods for predicting the cutting force components in face milling processes. Wang [23] discretized the end milling cutter along the circumferential direction and established an analytical cutting force model for the end milling cutter and simplified analytical indexes for predicting and estimating cutting force fluctuations. Kao [24] developed a mathematical model of the cutting forces of a ball-end milling cutter that includes tangential, radial, and axial cutting force components and characterized the relationship between average cutting force and feed per groove as a linear function. Kazuki [25] and Li [26] modeled the milling force of a ball-end milling cutter considering changes in the microelement friction angle, shear angle, and shear stress constraints using microelement diagonal shear theory. Falta [27] and Dong [28] used an analytical modeling method for predicting the static milling force of a ball-end milling cutter by establishing a tool–workpiece engagement region. The removal mechanism of materials can be deeply analyzed through the physical analytical model; however, the physical analytical model is closely related to material constitutive parameters, which are difficult to obtain. Therefore, the physical analytical model is not convenient for practical application, and its modeling process is complex and cumbersome.

A finite element model of milling force is a model that predicts milling forces and contact forces during the cutting process by discretizing the material regions being milled into small finite element regions. Soo [29,30] introduced a sophisticated finite element model developed for simulating the intricate process of high-speed ball nose end milling, specifically applied to Inconel 718, while successfully correlating predictions with experimental data. Filho [31] delved into the challenges posed by the low machinability of Inconel 718, and distinct plastic deformation levels between up and down milling, a factor attributed to the work-hardening behavior of Inconel 718, was revealed with finite element analysis and validated using experimental data. Hu [32] modeled the thermal-mechanical coupling property of ultrasonic torsional vibration-assisted micromilling (UTVAM) using ABAQUS software. Pratap [33] used the Johnson–Cook constitutive equation to develop a finite element model for microchannel micro-end milling of titanium alloys using ABAQUS that takes into account the strain, strain rate, and temperature effects on material properties. Molaiekiya [34] employed various methods, including tool life analysis, wear pattern investigation, and finite element analysis, to understand the behavior of SiAlON ceramic tools in high-speed face milling of Inconel 718. Deng [35] derived the milling mechanism of superalloy GH4169 under different processing parameters using the finite element method, and the results of the finite element analysis were successfully verified through milling experiment. Azeem [36] conducted molecular dynamics simulations to the investigate oxide–dislocation interaction mechanisms of oxide-dispersed strengthened (ODS) steel, and the interaction mechanisms between dislocations and oxides in ODS steel models were investigated. Azeem [37] utilized molecular dynamics (MD) simulations to gain insight into the irradiation effects on oxide-dispersed strengthened steel (ODSS) alloys, shedding light on the intricate microstructural changes and radiation resistance of these materials. Through using ABAQUS finite element software, Zhang [38] elucidated the impact of Al content on milling temperature, cutting forces, residual stress, and surface roughness during the milling process of CoCrFeNiAl_X_ high-entropy alloys. Bouzakis [39] introduced an innovative methodology for assessing the effectiveness of coated tools in milling challenging aerospace alloys; using analytical methods, impact tests, and finite element simulations, the study established a strong correlation between coating impact resistance and cutting performance under varying conditions. Kim [40] delved into the realm of the induction-assisted milling (IAM) of Inconel 718 and through the utilization of the Taguchi method and finite element analysis, the research identified optimal machining conditions for both coated and uncoated carbide tools. The finite element model of milling force can simulate the real milling process, consider the contact and interaction between the tool and the workpiece, and is able to predict the mechanical properties and deformation behavior of material. However, the use of different finite element models will lead to different accuracy results. Therefore, it is recommended that a higher precision, more targeted finite element model be chosen to simulate the milling process accurately and intuitively.

Chattering is an unstable state of motion in metal cutting processing, mainly caused by cutting force, cutting heat, and material deformation. Inconel 718 is difficult-to-machine material, and its high hardness and heat resistance make it more susceptible to chattering during the milling process. At present, the theoretical analysis methods for milling stability mainly include frequency domain analysis and time domain analysis.

Frequency domain analysis is a method used to evaluate and solve the chattering problem in the milling process by converting the dynamic response of the milling system to the frequency domain. The frequency domain analysis method can be further divided into the zero-order frequency domain method and the multifrequency method. Altintaş and Budak [41] proposed a zero-order frequency domain method of milling stability in which the oriented dynamic milling force coefficient matrix is transformed into the frequency domain by Fourier transform to solve for the stability of the system. Based on this, the critical axial depth of cut without causing chattering is determined. Merdol [42] considered the higher-order expansion terms of the Fourier series of the oriented dynamic milling force coefficient matrix and developed a multifrequency method that can be used in cases of different radial depths of cut.

Time domain analysis is another method used to evaluate and solve the chattering problem in the milling process by observing and analyzing the time response. The time domain analysis method can be further divided into the fully discrete and semi-discrete methods. Ding [43,44] verified the accuracy of the method using the single-degree-of-freedom and two-degree-of-freedom milling models and then proposed the second-order full-discrete method. Insperger [45,46,47] proposed a semi-discrete method to solve a system of time-delay differential equations containing a matrix of periodic coefficients. Subsequently, the method is improved so that it cannot only solve the stability limit of a single-degree-of-freedom vibration system but also a two-degree-of-freedom vibration system, and a first-order semi-discrete method with better convergence is proposed. Based on the semi-discrete method, Sun [48] established a stability lobe diagram of the rotary ultrasonic milling of titanium alloy by defining the ultrasonic function angle, and the results show that the rotary ultrasonic milling can suppress the machining vibration and improve the stability of the milling of titanium alloy. Gajera [49] proposed a study focusing on optimizing selective laser melting parameters for Invar-36 material, considering hardness and surface roughness as key performance indicators.

In summary, scholars have conducted research and a series of studies in the field of metal cutting and have achieved results in the modeling of milling forces and milling stability analysis. However, studies that concentrate on the milling mechanism and chattering stability of nickel-based superalloy Inconel 718 are relatively rare, and there exists no thorough and systematic research on the optimization of the processing parameters of nickel-based superalloy Inconel 718. Therefore, it is imperative to carry out in-depth research on the influence of different processing parameters on the milling of nickel-based superalloy Inconel 718 and the chattering stability during the milling process, as well as the optimization of the process parameters. In this paper, a milling force model of a ball-end milling cutter is established, and the milling force coefficients are deduced. According to the material failure criteria, a finite element model of both the tool and workpiece is created based on a finite element analysis, and the influence of different processing parameters on the milling of Inconel 718 is analyzed. Through applying the zero-order frequency domain method, a dynamic equation of the milling of Inconel 718 is established, and based on the modal experiment, stability lobe diagrams are drawn, which can be used as an essential reference for the selection of stable cutting parameters. Eventually, single factor experiments and orthogonal experiments are carried out to verify the correctness of the milling force model and results of the finite element analysis. After the reliability of the milling force model and results of the finite element analysis is demonstrated, the fmincon optimization algorithm is used to optimize the processing parameters of Inconel 718, and the best processing parameters under different processing requirements are determined.

This article presents a novel exploration of the machining of nickel-based superalloy Inconel 718, addressing a significant gap in the existing literature. While studies in metal cutting and machining are prevalent, there is a distinct lack of comprehensive research concerning the milling mechanism and chattering stability of Inconel 718. A distinct milling force model is formulated for ball-end milling cutters applied specifically to Inconel 718. This model enables the precise comprehension of the forces involved during machining, enhancing insights into the effects of different parameters. Incorporating finite element analysis, the intricate relationship among various processing parameters and the material behavior of Inconel 718 during machining are explored. A novel aspect of this work is the meticulous examination of the chattering stability during the machining process. This exploration is paramount to achieving stable machining outcomes for challenging materials like Inconel 718 so that the appropriate processing parameters can be selected to prevent chattering. Lastly, this study introduces a multi-objective optimization strategy, where conflicting objectives, such as surface roughness and material removal rate, are concurrently considered. Based on the multi-objective optimization algorithm, the best machining parameters are determined, which point to an improvement in the machining efficiency and quality of Inconel 718, which has not been addressed by current studies.

## 2. Modeling of Milling Forces

### 2.1. Modeling of Milling Forces of Ball-End Milling Cutter

**Figure 1:**
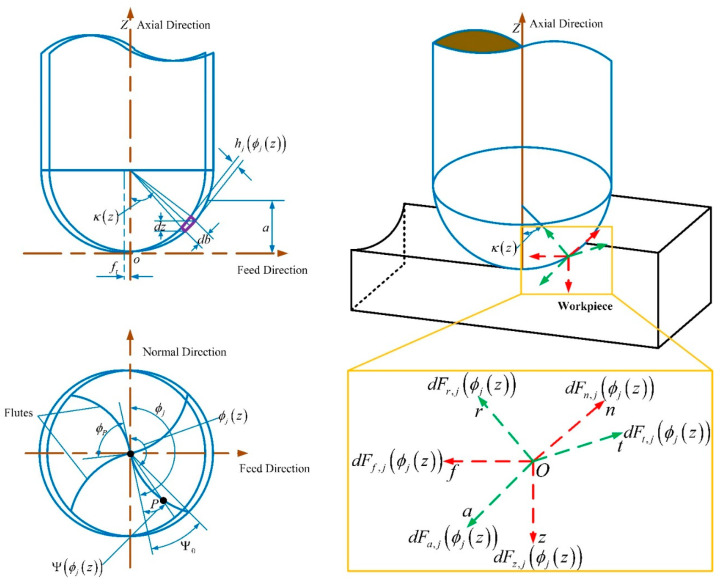
Milling force model of ball-end milling cutter.

The milling state of the helix cutting edge of a ball-end milling cutter varies with different cutting positions. Thus, it is essential to develop a geometric model of the ball-end milling cutter to investigate the change in the milling force when the cutting edge interacts with the workpiece. The schematic of the proposed model is illustrated in Figure 1.

According to the literature [24], neglecting the tip radius of the ball-end milling cutter, the cutting force on any microelement of the cutting edge can be described using Equation (1).

(1) $$\left\{\begin{array}{l} dF_{t,j}\left(\phi_{j}\left(z\right)\right)=K_{te}dS\left(\phi_{j}\left(z\right)\right)+K_{tc}h_{j}\left(\phi_{j}\left(z\right)\right)db\\ dF_{r,j}\left(\phi_{j}\left(z\right)\right)=K_{re}dS\left(\phi_{j}\left(z\right)\right)+K_{rc}h_{j}\left(\phi_{j}\left(z\right)\right)db\\ dF_{a,j}\left(\phi_{j}\left(z\right)\right)=K_{ae}dS\left(\phi_{j}\left(z\right)\right)+K_{ac}h_{j}\left(\phi_{j}\left(z\right)\right)db \end{array}\right.$$

The instantaneous chip thickness at the immersion angle $h_{j}\left(\phi_{j}\left(z\right)\right)$ can be expressed as:

(2) $$h_{j}\left(\phi_{j}\left(z\right)\right)=f_{t}\sin\left(\phi_{j}\left(z\right)\right)\sin\left(\kappa \left(z\right)\right).$$

The instantaneous edge length of the cutting segment $dS\left(\phi_{j}\left(z\right)\right)$ can be expressed as:

(3) $$dS=dz\cdot \sqrt{\frac{{\left(R_{0}-z\right)}^{2}}{R_{0}{}^{2}-{\left(R_{0}-z\right)}^{2}}+\frac{\tan^{2}\beta }{R_{0}{}^{2}}\left[R_{0}{}^{2}-{\left(R_{0}-z\right)}^{2}\right]+1}=M\cdot dz$$

The instantaneous chip width db can be expressed as:

(4) $$db=\frac{dz}{\sin\left(\kappa \left(z\right)\right)}=\frac{R_{0}}{\sqrt{R_{0}{}^{2}-{\left(R_{0}-z\right)}^{2}}}dz$$

By transforming the cutting force microelement from coordinate system O−tra to O−fnz and integrating it, the cutting force of the milling cutter along the feed, normal, and axial directions can be obtained as:

(5) $$\left[\begin{array}{c} F_{f}\left(\phi \right)\\ F_{n}\left(\phi \right)\\ F_{z}\left(\phi \right) \end{array}\right]=\left[\begin{array}{c} \sum_{j=1}^{N}F_{f,j}\left(\phi_{j}\right)\\ \sum_{j=1}^{N}F_{n,j}\left(\phi_{j}\right)\\ \sum_{j=1}^{N}F_{z,j}\left(\phi_{j}\right) \end{array}\right]$$

Subsequently, the average milling force per rotation of the ball-end milling cutter can be calculated using the Equation (6):

(6) $$\left\{\begin{array}{l} \bar{F_{f}}=\frac{N_{f}}{2\pi }\int_{0}^{2\pi }\left(\int_{z_{1}\left(\phi_{j}\right)}^{z_{2}\left(\phi_{j}\right)}dF_{f}\left(\phi_{j}\left(z\right)\right)\right)d\phi \\ \bar{F_{n}}=\frac{N_{f}}{2\pi }\int_{0}^{2\pi }\left(\int_{z_{1}\left(\phi_{j}\right)}^{z_{2}\left(\phi_{j}\right)}dF_{n}\left(\phi_{j}\left(z\right)\right)\right)d\phi \\ \bar{F_{z}}=\frac{N_{f}}{2\pi }\int_{0}^{2\pi }\left(\int_{z_{1}\left(\phi_{j}\right)}^{z_{2}\left(\phi_{j}\right)}dF_{z}\left(\phi_{j}\left(z\right)\right)\right)d\phi \end{array}\right.$$

Equation (7) is finally obtained by simplifying the equation of the microelement of the milling force and substituting it into Equation (6):

(7) $$\left\{\begin{array}{l} {\bar{F}}_{f}=\left(C_{1}K_{tc}+C_{3}K_{rc}+C_{5}K_{ac}\right)f_{t}+C_{2}K_{te}+C_{4}K_{re}+C_{6}K_{ae}\\ {\bar{F}}_{n}=\left(C_{7}K_{tc}+C_{9}K_{rc}+C_{11}K_{ac}\right)f_{t}+C_{8}K_{te}+C_{10}K_{re}+C_{12}K_{ae}\\ {\bar{F}}_{z}=\left(C_{13}K_{rc}+C_{15}K_{ac}\right)f_{t}+C_{14}K_{re}+C_{16}K_{ae} \end{array}\right.$$

The parameters $C_{1}-C_{16}$ in Equation (7) were provided in Reference [24].

### 2.2. Recognition Experiment of Milling Force Coefficients

In order to accurately determine the milling force generated during milling processing, it is necessary to determine the milling force coefficients $K_{te}$, $K_{re}$, $K_{ae}$, $K_{tc}$, $K_{rc}$, and $K_{ac}$ in Equation (7); therefore, recognition experiments of the milling force coefficients are required.

For this experiment, the 1B240-0800-XA 1630 carbide ball-end milling cutter (developed by Sandvik in Germany) was chosen, the diameter of the cutter was 8 mm, the overall length of the cutter was 100 m, the depth of cut was set to 0.5 mm, and the rotating speed of spindle was set to 1000 rpm. To facilitate the calculation of the milling force coefficients, slot milling was used as the milling method, and the selected machining parameters and the measured average milling force data are recorded in Table 1.

Based on the data in Table 1, the milling force coefficients were obtained using the coefficient recognition equation of the milling force (Equation (8)).

The coefficients of the milling force calculating through Equation (8) are represented in Table 2.


(8) $$\left\{\begin{array}{l} K_{ac}=\frac{{\bar{F}}_{zc}\left(C_{3}C_{7}C_{15}-C_{1}C_{9}C_{15}\right)-C_{7}C_{13}C_{15}{\bar{F}}_{fc}+C_{1}C_{13}C_{15}{\bar{F}}_{nc}}{C_{3}C_{7}C^{2}{}_{15}-C_{5}C_{7}C_{13}C_{15}-C_{1}C_{9}C^{2}{}_{15}+C_{1}C_{11}C_{13}C_{15}}\\ K_{ae}=\frac{{\bar{F}}_{ze}\left(C_{4}C_{8}C_{16}-C_{2}C_{10}C_{16}\right)-C_{14}C_{8}C_{16}{\bar{F}}_{fe}+C_{2}C_{14}C_{16}{\bar{F}}_{ne}}{C_{4}C_{8}C^{2}{}_{16}-C_{6}C_{8}C_{14}C_{16}-C_{2}C_{10}C^{2}{}_{16}+C_{2}C_{12}C_{14}C_{16}}\\ K_{rc}=\frac{C_{7}C_{15}{\bar{F}}_{fc}-C_{1}C_{15}{\bar{F}}_{nc}+\left(C_{1}C_{11}-C_{5}C_{7}\right){\bar{F}}_{zc}}{C_{3}C_{7}C_{15}-C_{5}C_{7}C_{13}-C_{1}C_{9}C_{15}+C_{1}C_{11}C_{13}}\\ K_{re}=\frac{C_{8}C_{16}{\bar{F}}_{fe}-C_{2}C_{16}{\bar{F}}_{ne}+\left(C_{2}C_{12}-C_{8}C_{6}\right){\bar{F}}_{ze}}{C_{4}C_{8}C_{16}-C_{6}C_{8}C_{14}-C_{2}C_{10}C_{16}+C_{2}C_{12}C_{14}}\\ K_{tc}=\frac{{\bar{F}}_{fc}-C_{3}K_{rc}-C_{5}K_{zc}}{C_{1}}\\ K_{te}=\frac{{\bar{F}}_{fe}-C_{4}K_{re}-C_{6}K_{ze}}{C_{2}} \end{array}\right.$$

## 3. Finite Element Analysis of Milling of Inconel 718

**Figure 2:**
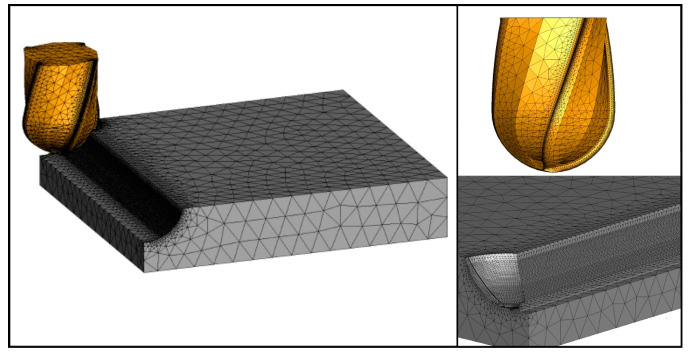
Finite element model of tool and workpiece.

In this study, a finite element model is established, as shown in Figure 2, including the workpiece and the tool. In the following, the temperature, stress, and milling force in the milling process are calculated. Finally, the influence of different machining parameters on the machining condition of Inconel 718 is evaluated through simulation results.

### 3.1. Methodology of Finite Element Analysis

Nickel-based superalloy Inconel 718 was chosen as the research object of the finite element analysis, and AdvantEdge (version 7.1, developed by Third Wave System in America) was utilized as the analysis software.

To study the effect of different processing parameters, such as spindle speed, feed per tooth, and radial and axial depths of cut, on the temperature, stress, and cutting force generated during the milling process of Inconel 718, the parameters shown in Table 3 were used for the finite element analysis.

The constitutive model is a critical prerequisite of the accuracy and reliability of the finite element analysis. The behavior of materials and results of an engineering problem can be better provided by the right chosen constitutive model [50]. The constitutive models applicable to metal cutting are mainly the power law and Johnson–Cook models [51]. The power law constitutive model is usually applicable to the analysis of the deformation behavior of materials at high temperatures and high strain rates, while the Johnson–Cook constitutive model is more suitable for the analysis of the deformation behavior of materials at low to medium temperatures and low to medium strain rates. Therefore, the deformation behavior of Inconel 718 at high temperatures and high strain rates can be described using the power law constitutive model. The mathematical equation of the power law model can be expressed as:

(9) $$\sigma \left(\varepsilon^{p},\dot{\varepsilon },T\right)=g\left(\varepsilon^{p}\right)\Gamma (\dot{\varepsilon })\Theta (T)$$

In Equation (9), the strain enhancement function $g\left(\varepsilon^{p}\right)$ can be further expressed as:

(10) $$\left\{\begin{array}{ll} g\left(\varepsilon^{p}\right)=\sigma_{0}{\left(1+\frac{\varepsilon^{p}}{\varepsilon_{0}^{p}}\right)}^{1/n} & \varepsilon^{p}<\varepsilon_{cut}^{p}\\ g\left(\varepsilon^{p}\right)=\sigma_{0}{\left(1+\frac{\varepsilon_{cut}^{p}}{\varepsilon_{0}^{p}}\right)}^{1/n} & \varepsilon^{p}\geq \varepsilon_{cut}^{p} \end{array}\right.$$

The strain enhancement parameters of Inconel 718 are shown in Table 4.

In Equation (9), the thermal softening function $\Theta \left(T\right)$ can be expressed as:

(11) $$\left\{\begin{array}{ll} \Theta (T)=c_{0}+c_{1}T+c_{2}T^{2}+c_{3}T^{3}+c_{4}T^{4}+c_{5}T^{6} & T<T_{\mathrm{cut}\;}\\ \Theta (T)=\Theta \left(T_{\mathrm{cut}\;}\right)\left(1-\frac{T-T_{\mathrm{cut}\;}}{T_{\mathrm{melt}\;}-T_{\mathrm{cut}\;}}\right) & T\geq T_{\mathrm{cut}} \end{array}\right.$$

The thermal softening parameters of Inconel 718 are shown in Table 5.

In Equation (9), the strain rate effect function $\Gamma \left(\dot{\varepsilon }\right)$ can be expressed as:

(12) $$\left\{\begin{array}{ll} \Gamma (\dot{\varepsilon })={\left(1+\frac{\dot{\varepsilon }}{{\dot{\varepsilon }}_{0}}\right)}^{\frac{1}{m_{1}}} & \dot{\varepsilon }\leq {\dot{\varepsilon }}_{t}\\ \Gamma (\dot{\varepsilon })={\left(1+\frac{\dot{\varepsilon }}{{\dot{\varepsilon }}_{0}}\right)}^{\frac{1}{m_{2}}}{\left(1+\frac{{\dot{\varepsilon }}_{t}}{{\dot{\varepsilon }}_{0}}\right)}^{\left(\frac{1}{m_{1}}-\frac{1}{m_{2}}\right)} & \dot{\varepsilon }>{\dot{\varepsilon }}_{t} \end{array}\right.$$

The strain rate parameters of Inconel 718 are shown in Table 6.

The damage model for Inconel 718 can be determined using the following equation:

(13) $$D=\sum_{i}\frac{\Delta \varepsilon_{i}^{p}}{\varepsilon_{f_{i}}^{p}}$$

In Equation (13), the parameter $\Delta \varepsilon_{i}^{p}$ can be expressed as:

(14) $$\varepsilon_{f_{0}}^{p}=d_{0}+d_{1}T+d_{2}T^{2}+d_{3}T^{3}+d_{4}T^{4}+d_{5}T^{6}$$

In Equation (14), the parameters $d_{0}-d_{5}$ are the strain values deduced using the tensile test.

### 3.2. Result of Finite Element Analysis

#### 3.2.1. Temperature Field Analysis of Milling Process

Inconel 718 is a difficult-to-machine material, which has low thermal conductivity. The phenomenon of thermal mechanical coupling could occur during the machining process, which results in residual stress and reduces the surface quality. Therefore, to effectively control tool wear and improve the surface integrity of the workpiece, the milling temperatures must be systematically studied and analyzed.

**Figure 3:**
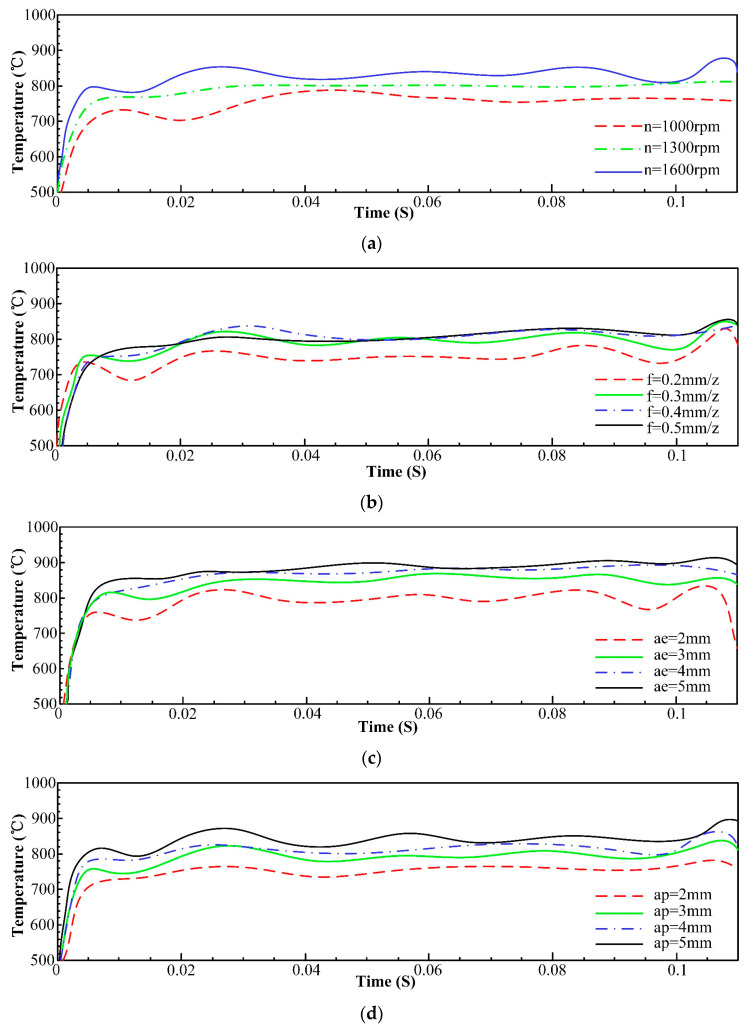
Influence of processing parameters on temperature. (**a**) Influence of spindle speed on temperature; (**b**) Influence of feed per tooth on temperature; (**c**) Influence of radial depth of cut on temperature; (**d**) Influence of axial depth of cut on temperature.

The fitted curves of the maximum temperature that occurred at the tool–workpiece contact region at different spindle speeds, feed per tooth, and radial and axial depths of cut are plotted according to the results of the finite element analysis, and these curves are shown in Figure 3a–d.

As shown in Figure 3, the temperature increases as the spindle speed rises, and this is mainly due to the fact that the friction phenomenon between the front (or back) of the tool and workpiece becomes more intense as the spindle speed increases, and this increasing trend is especially conspicuous near the cutting edge. Moreover, with the increase in the radial depth of cut, axial depth of cut, and feed per tooth, the temperature also increases, because increases in all of these parameters cause the tool to cut more material per unit of time, so the heat continues to accumulate and more heat diffuses into the tool.

#### 3.2.2. Stress Field Analysis of Milling Process

Because of the high hardness and strength properties of Inconel 718, the milling process of this material is susceptible to stress concentration and crack formation. Stress field simulation enables the visualization of the stress distribution on both the tool and workpiece surfaces during machining, facilitating a deeper understanding of the physical phenomena involved. Consequently, the impact of various machining parameters on stress can be predicted to mitigate stress concentration and prevent crack formation.

**Figure 4:**
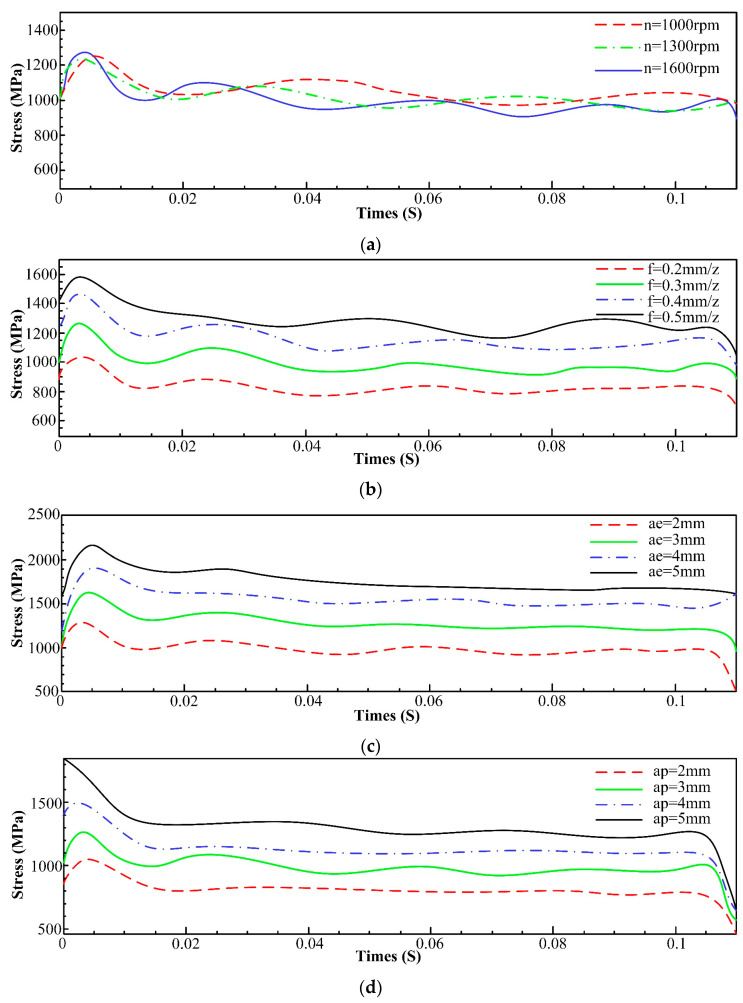
Influence of machining parameters on stress. (**a**) Influence of spindle speed on stress; (**b**) Influence of feed per tooth on stress; (**c**) Influence of radial depth of cut on stress; (**d**) Influence of axial depth of cut on stress.

To investigate the impact of processing parameters on stresses during machining, the stress values in the top 10% of elements with the highest stresses on the tool are averaged and plotted as a function of time. Fitted curves representing stress variations at different spindle speeds, feed per tooth, and radial and axial depths of cut are presented in Figure 4a–d.

From Figure 4, it is evident that an increase in the spindle speed does not significantly affect the stress, as the material volume cut by the tool remains unchanged despite the enlargement of the spindle speed. Conversely, with an increment in feed per tooth, radial depth of cut, and axial depth of cut, there is a simultaneous increase in stress. This observation can be attributed to the larger volume of material being removed because of changes in these parameters. Therefore, more force is required to remove the material from the surface of the workpiece, and the stress acting on the tool tends to increase.

#### 3.2.3. Force Analysis of Milling Process

Given that the magnitude of the milling force directly influences the wear and fracture condition of the tool, a comprehensive analysis of the milling force generated during the Inconel 718 milling process becomes crucial.

**Figure 5:**
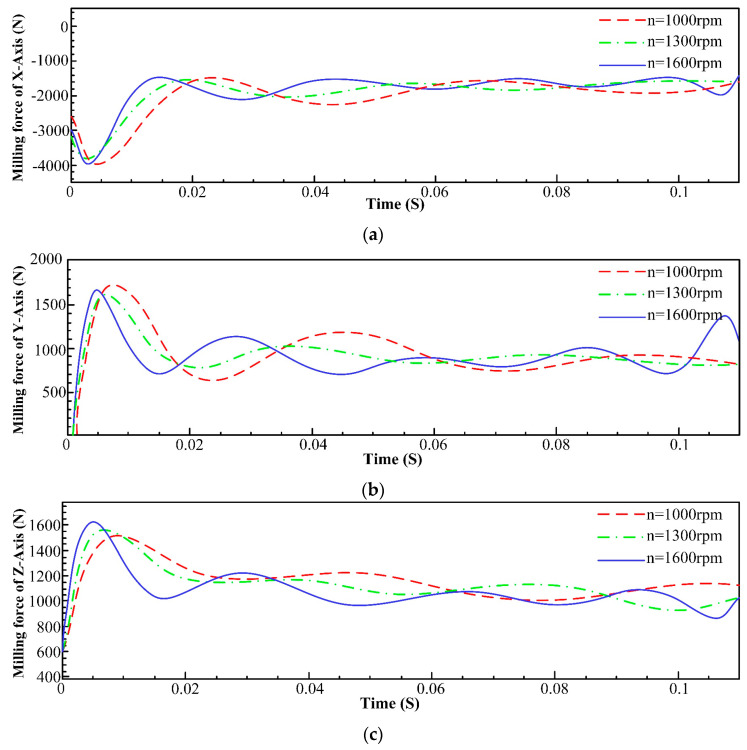
Comparison of milling forces of each axis at different spindle speeds. (**a**) Influence of spindle speed on milling force of x-axis; (**b**) Influence of spindle speed on milling force of y-axis; (**c**) Influence of spindle speed on milling force of z-axis.

In order to better study the influence trend of different machining parameters on milling force, the spindle speed was set to 1000 rpm, 1300 rpm, and 1600 rpm, and the other parameters were kept unchanged. The milling force data of each axis are shown in Figure 5.

Figure 5 illustrates that the effect of different spindle speeds on the magnitude of the milling force for each axis is not significant. This can be attributed to the nearly constant volume of material being removed, regardless of the spindle speed variation. Actually, Inconel 718 is known for its high toughness and work-hardening characteristics. As the spindle speed increases, the cutting edge may experience slightly different interactions with the material due to the variations in chip thickness and heat generation. However, the material’s inherent properties, including its resistance to deformation and heat, might minimize the impact of spindle speed changes on the cutting forces.

**Figure 6:**
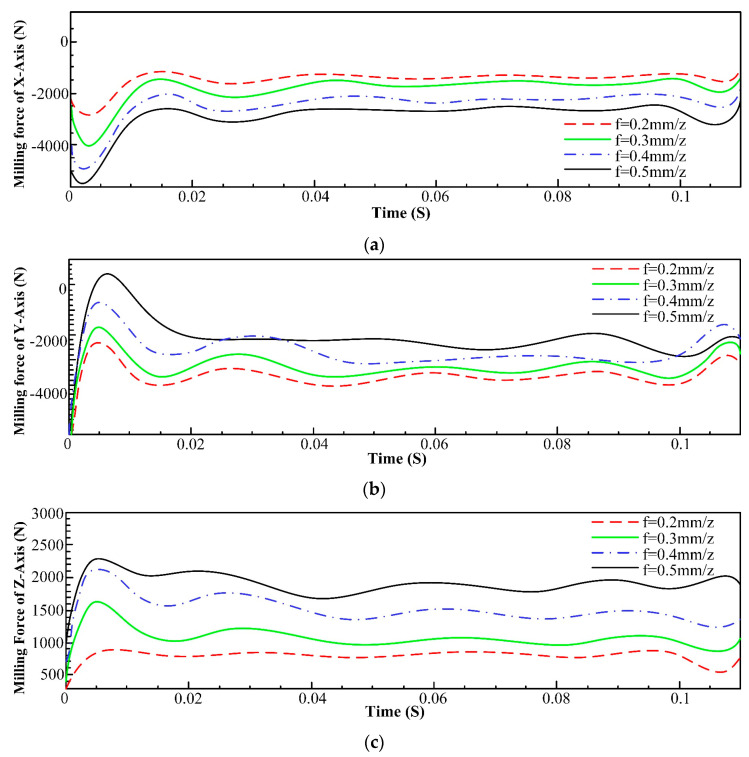
Comparison of milling forces of each axis at different feeds per tooth. (**a**) Influence of feed per tooth on milling force of x-axis; (**b**) Influence of feed per tooth on milling force of y-axis; (**c**) Influence of feed per tooth on milling force of z-axis.

The feed per tooth was varied at values of 0.2 mm, 0.3 mm, 0.4 mm, and 0.5 mm while keeping the remaining parameters unchanged. The corresponding milling force data for each axis are presented in Figure 6.

Figure 6 demonstrates a clear and consistent increasing trend in the milling forces for each axis with the increase in the feed per tooth. This observation can be attributed to the larger volume of workpiece material being removed by the cutter as the feed per tooth increases, because Inconel 718 is known for its high strength, toughness, and resistance to deformation. As the feed per tooth increases, more material is being removed with each tooth engagement. This requires the cutting tool to exert greater force to cut through the tough material. When machining Inconel 718, heat generated at the cutting interface can influence the material’s behavior. Higher feeds can lead to more heat being generated due to increased friction, and this can affect the material’s strength and work-hardening tendencies, potentially increasing the forces needed to cut through the material. The chip formation also accounts for this phenomenon of increasing milling force, since the increase in feed per tooth can lead to the generation of larger chips or greater chip thickness. Inconel 718 tends to form short, tightly curled chips that can exert backpressure on the cutting tool, causing additional cutting forces.

**Figure 7:**
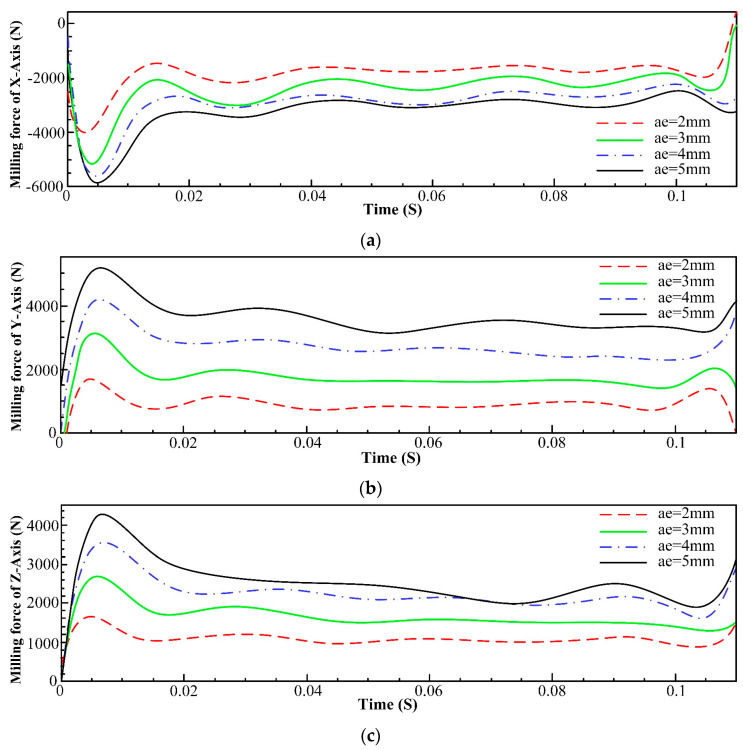
Comparison of milling forces of various axes under different radial depths of cut. (**a**) Influence of radial depth of cut on milling force of x-axis; (**b**) Influence of radial depth of cut on milling force of y-axis; (**c**) Influence of radial depth of cut on milling force of z-axis.

The radial depth of cut was varied at values of 2 mm, 3 mm, 4 mm, and 5 mm while keeping the remaining parameters unchanged. The corresponding milling force data for each axis are presented in Figure 7.

Figure 7 reveals that the milling forces in the x-axis, y-axis, and z-axis exhibit a notable increase with the rise in the radial depth of cut. A larger radial depth of cut means that a larger portion of the cutting edge is engaged with the material. This results in a larger contact area between the tool and the workpiece. Therefore, the tool has to remove material from a wider area, requiring more force to achieve this. Inconel 718 has high strength and hardness, which means that cutting through it requires more force. In addition, as the cutting tool engages with the material, it generates heat due to the friction. This heat can cause work hardening in the material, making it even tougher to cut. With a higher radial depth of cut, more heat is generated, leading to increased work hardening and subsequently higher cutting forces. Apart from this, Inconel 718 tends to produce shorter, more tightly curled chips because of its toughness and heat resistance. These chips can put additional stress on the cutting tool and increase the cutting forces. With a higher radial depth of cut, there is more material being removed and, consequently, more chips are generated, contributing to higher forces. In addition, cutting forces can also increase because of accelerated tool wear. The high temperatures generated during the machining process, especially with a higher radial depth of cut, can lead to faster tool wear. As the tool wears, it becomes less effective, requiring higher forces to achieve the same level of material removal.

**Figure 8:**
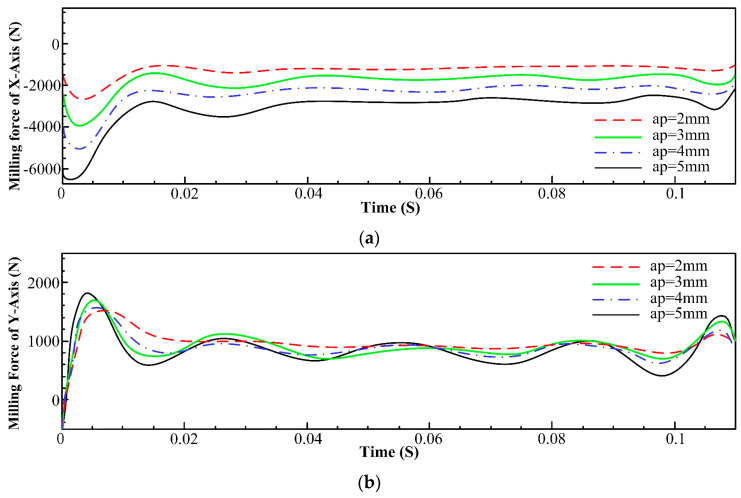

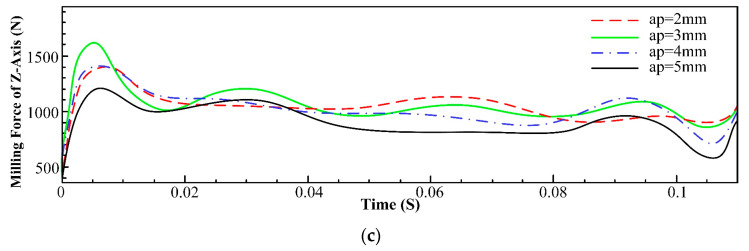
Comparison of the milling forces of different axes under different axial depths of cut. (**a**) Influence of axial depth of cut on milling force of x-axis; (**b**) Influence of axial depth of cut on milling force of y-axis; (**c**) Influence of axial depth of cut on milling force of z-axis.

Similarly, the value of the axial depth of cut was set to 2 mm, 3 mm, 4 mm, and 5 mm, and the rest of the parameters were kept unchanged; the milling force data of each axis are shown in Figure 8. The data shown in Figure 8a unveil that the milling forces of the x-axis increase with an augmentation of the value of the axial depth of cut. This phenomenon can be explained by the abovementioned reasons. Figure 8b,c disclose that the milling forces in the y- and z-axes do not show a tangible rising tendency with the increase in the radial depth of cut. This observation can be attributed to the fact that a higher radial depth of cut primarily affects the x-axis (radial) direction forces, while the y and z forces are influenced by the tool’s interaction with the material in their respective directions. If the tool’s engagement angle remains relatively consistent, it might lead to stable forces. Furthermore, in the y- and z-directions, the material might flow more smoothly as the tool progresses through the cut. This controlled material flow can lead to more predictable forces, even with variations in the radial depth of cut.

## 4. Analysis of Milling Stability

### 4.1. Establishment of Dynamic Equation for Milling Inconel 718

Inconel 718 belongs to difficult-to-machine materials and is susceptible to the phenomenon of chattering during the milling process. Chattering in the milling process will lead to a positive feedback effect between the cutting force and vibration, which will result in degradation of the milling quality, cutting edge wear, tool fracture, and other problems. Through a stability analysis of the chattering, the stability condition of the milling process can be predicted, and whether chattering will occur during the milling process can also be determined. Therefore, it is necessary to carry out a chattering stability analysis of Inconel 718 to improve the milling efficiency and machining quality, as well as to reduce the production cost.

**Figure 9:**
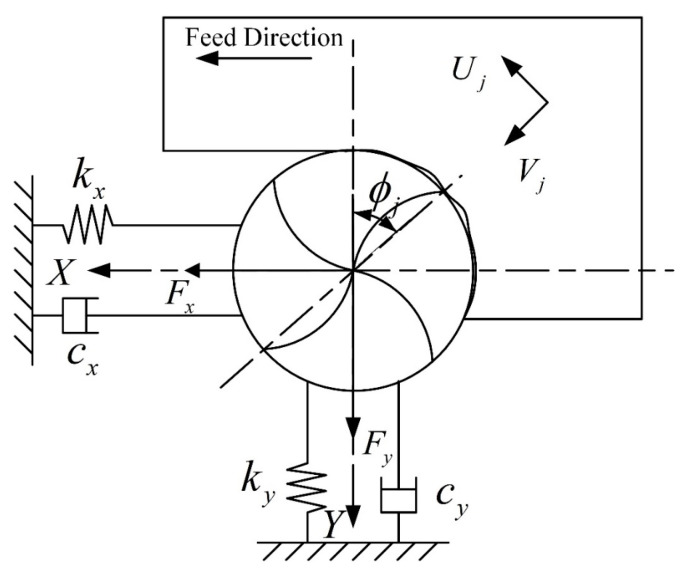
Dynamic model of the machine–tool system.

As shown in Figure 9, the machine–tool system is simplified as a vibration system with two degrees of freedom, where the vibrations in the x- and y-directions can be described using the kinetic differential equations:

(15) $$\left\{\begin{array}{l} m_{x}\ddot{x}+c_{x}\dot{x}+k_{x}x=\sum_{j=1}^{N}F_{xj}=F_{x}(t)\\ m_{y}\ddot{y}+c_{y}\dot{y}+k_{y}y=\sum_{j=1}^{N}F_{yj}=F_{y}(t) \end{array}\right.$$

**Figure 10:**
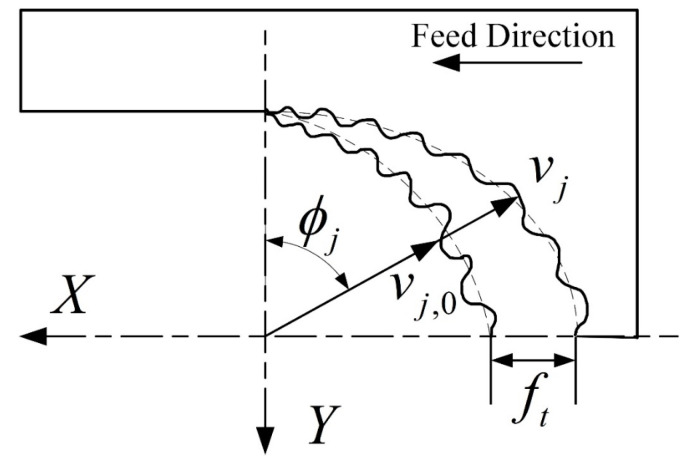
Dynamic change in the cutting thickness.

In the milling process, as shown in Figure 10, the current cutting process will be affected by the previous cutting process, since the previous machining process leaves vibrations on the machined surface, and when the tool cuts the surface again, this can lead to changes in the cutting thickness. Such changes in the cutting thickness can cause fluctuations in the cutting force, leading to further vibrations in the system. The total cutting thickness generated by the superposition of the static cutting thickness and the dynamic cutting thickness caused by the tool vibration can be expressed as:

(16) $$h\left(\phi_{j}\right)=\left[f_{t}\sin\phi_{j}+\left(v_{j,0}-v_{j}\right)\right]g\left(\phi_{j}\right)$$

In Equation (16), the parameter $g\left(\phi_{j}\right)$ is a unit step function, which is expressed below:

(17) $$g\left(\phi_{j}\right)=\left\{\begin{array}{l} 1\;,\;\left(\phi_{st}<\phi_{j}<\phi_{ex}\right)\\ 0,\;\left(\phi_{j}<\phi_{st},\phi_{j}>\phi_{ex}\right) \end{array}\right.$$

Therefore, the total cutting force on the cutter can be expressed as:

(18) $$\left\{\begin{array}{c} F_{x}\\ F_{y} \end{array}\right\}=\frac{1}{2}a_{p}K_{tc}\left[\begin{array}{cc} \alpha_{xx} & \alpha_{xy}\\ \alpha_{yx} & \alpha_{yy} \end{array}\right]\left\{\begin{array}{c} \Delta x\\ \Delta y \end{array}\right\}$$

In Equation (18), the parameters $\alpha_{xx}$, $\alpha_{xy}$, $\alpha_{yx}$, and $\alpha_{yy}$ are the average directional force coefficient:

(19) $$\left\{\begin{array}{l} \alpha_{xx}=\sum_{j=0}^{N-1}-g_{j}\left[\sin2\phi_{j}+K_{r}\left(1-\cos2\phi_{j}\right)\right]\\ \alpha_{xy}=\sum_{j=0}^{N-1}-g_{j}\left[\left(1+\cos2\phi_{j}\right)+K_{r}\sin2\phi_{j}\right]\\ \alpha_{yx}=\sum_{j=0}^{N-1}g_{j}\left[\left(1-\cos2\phi_{j}\right)+K_{r}\sin2\phi_{j}\right]\\ \alpha_{yy}=\sum_{j=0}^{N-1}g_{j}\left[\sin2\phi_{j}-K_{r}\left(1+\cos2\phi_{j}\right)\right] \end{array}\right.$$

Thus, the equation of the dynamic milling force can be expressed as:

(20) $$\left\{F\left(t\right)\right\}=\frac{1}{2}a_{p}K_{tc}\left[A\left(t\right)\right]\left\{\Delta \left(t\right)\right\}$$

Equation (20) can be further simplified as:

(21) $$\left\{F\left(t\right)\right\}=\frac{1}{2}a_{p}K_{tc}\left[A_{0}\right]\left\{\Delta \left(t\right)\right\}$$

In Equation (21), the parameter $\left[A_{0}\right]$ can be expressed as:

(22) $$\left[A_{0}\right]=\frac{1}{T}\int_{0}^{T}\left[A\left(t\right)\right]dt=\frac{1}{\phi_{p}}\int_{\phi_{st}}^{\phi_{ex}}\left[A\left(\phi \right)\right]d\phi =\frac{N}{2\pi }\left[\begin{array}{cc} \alpha_{xx} & \alpha_{xy}\\ \alpha_{yx} & \alpha_{yy} \end{array}\right]$$

The stability of the system is solved using the zero-order frequency domain method, such that the frequency response transfer function matrix of the tool–workpiece contact zone is:

(23) $$\left[\Phi \left(i\omega \right)\right]=\left[\begin{array}{cc} \Phi_{xx}\left(i\omega \right) & \Phi_{xy}\left(i\omega \right)\\ \Phi_{yx}\left(i\omega \right) & \Phi_{yy}\left(i\omega \right) \end{array}\right]$$

### 4.2. Methodology of the Modal Experiment

To solve the dynamic equation, it is necessary to obtain the modal parameters of the tool, including the natural frequency, damping ratio, and vibration mode. The approach of the force hammer excitation was used in this experiment to obtain accurate modal parameters.

**Figure 11:**
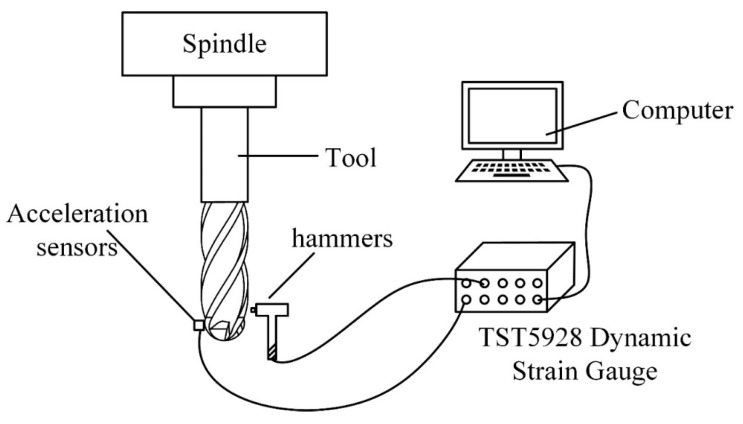
Schematic diagram of the equipment connection of the modal experiment.

**Figure 12:**
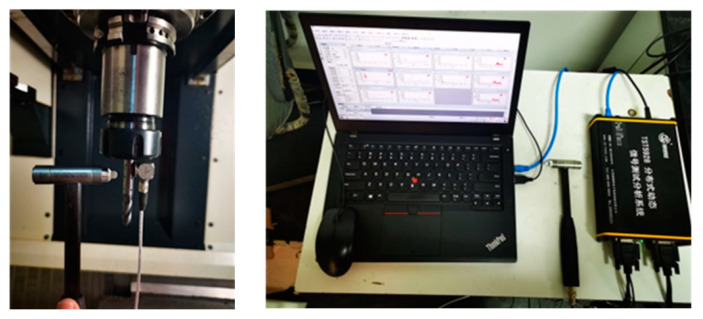
Equipment connection for the modal experiment.

The equipment utilized for conducting the modal experiment included a 1B240-0800-XA carbide ball-end milling cutter (developed by Sandvik in Germany), a three-way acceleration sensor (developed by Kistler in Switzerland), a TST5928 Dynamic Strain Gauge, and a computer equipped with the TST5928 Distributed Dynamic Signal Test and Analysis System. A schematic of the hardware installation of the system for the modal experiment is shown in Figure 11, and photos of the connection of the experimental equipment is shown in Figure 12.

**Figure 13:**
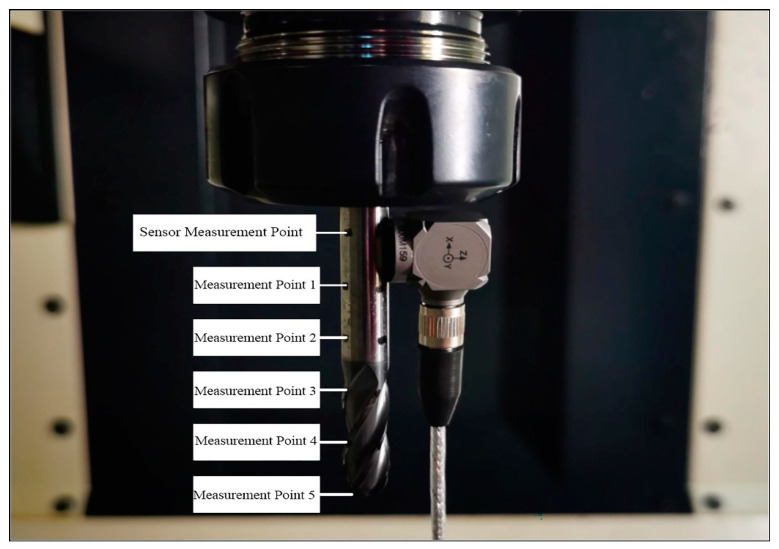
Distribution of measuring points on tool.

Figure 13 shows the distribution of the measurement points of the tool, where a force hammer was used to excite the tool at different measurement points, and the response of the tool was measured. Since the stiffness of the tool in the z-direction was much greater than in the x- and y-directions, only modal experiments in the x- and y-directions were performed.

### 4.3. The Result of Modal Experiment

The results of modal experiments are recorded in Table 7.

**Figure 14:**
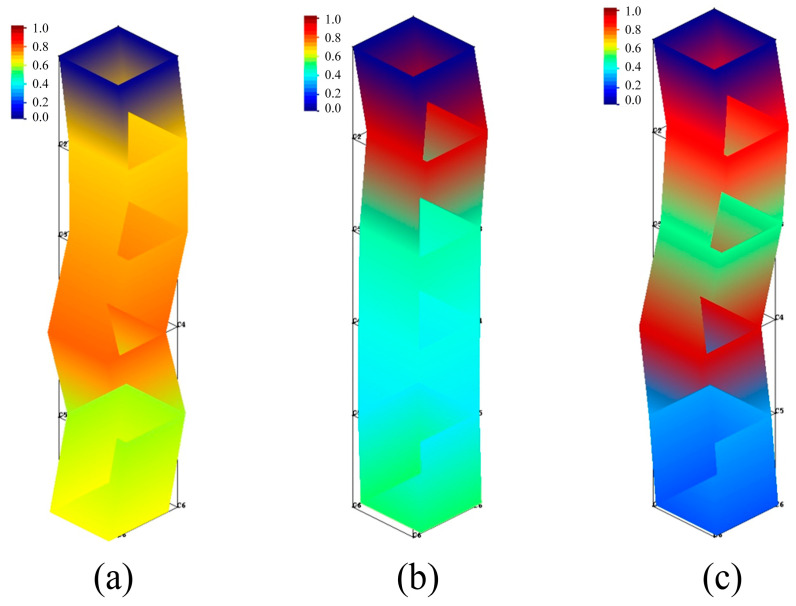
Tool modal of x-direction. (**a**) First order of x-direction; (**b**) Second order of x-direction; (**c**) Third order of x-direction.

**Figure 15:**
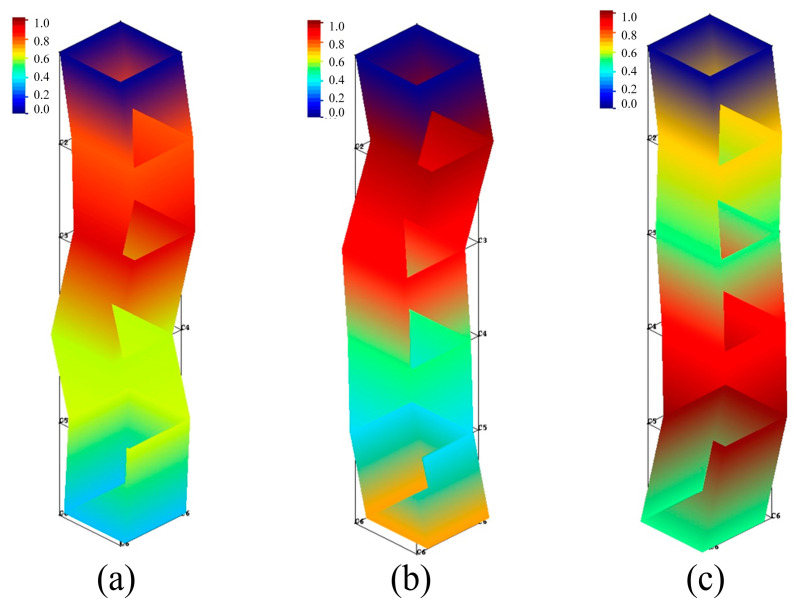
Tool modal of the y-direction. (**a**) First order of y-direction; (**b**) Second order of y-direction; (**c**) Third order of y-direction.

Figure 14 and Figure 15 show the tool modal of the x- and y-directions, respectively.

Combining the results of Figure 14 and Figure 15, as well as Table 7, it is possible to derive that the modal shapes corresponding to the first-order natural frequency in the x- and y-directions of the tool are both bent and deformed in the middle part of the tool. The modal shapes corresponding to the second-order natural frequency in the x- and y-directions of the tool are both bent and deformed in the upper part of the tool, and the degree of deformation in the y-direction is slightly larger than that in the x-direction. The bending deformation of the modal shapes corresponding to third-order natural frequency in the x-direction occurs in the middle and upper parts of the tool, and the deformation of the bottom of the tool is small, while the bending deformation of the modal shapes corresponding to the third-order natural frequency in the y-direction occurs in the lower part of the tool.

**Figure 16:**
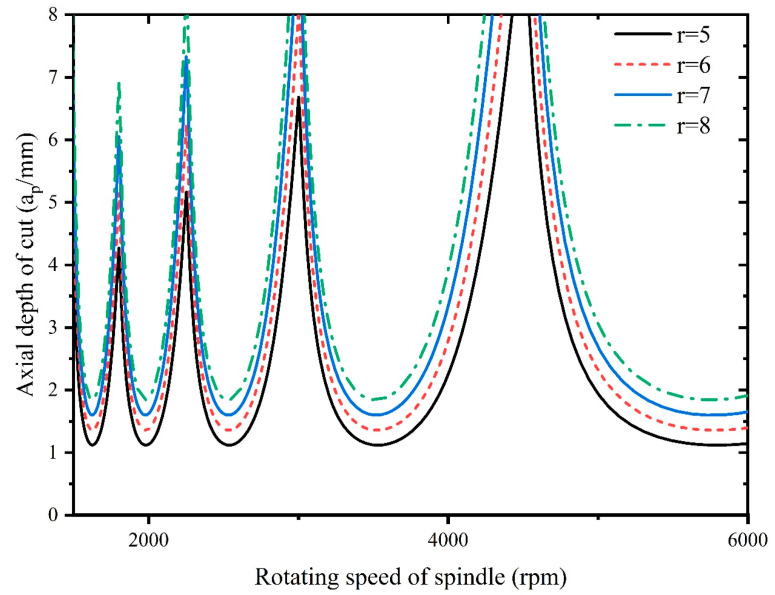
Influence of tool radius on stability.

**Figure 17:**
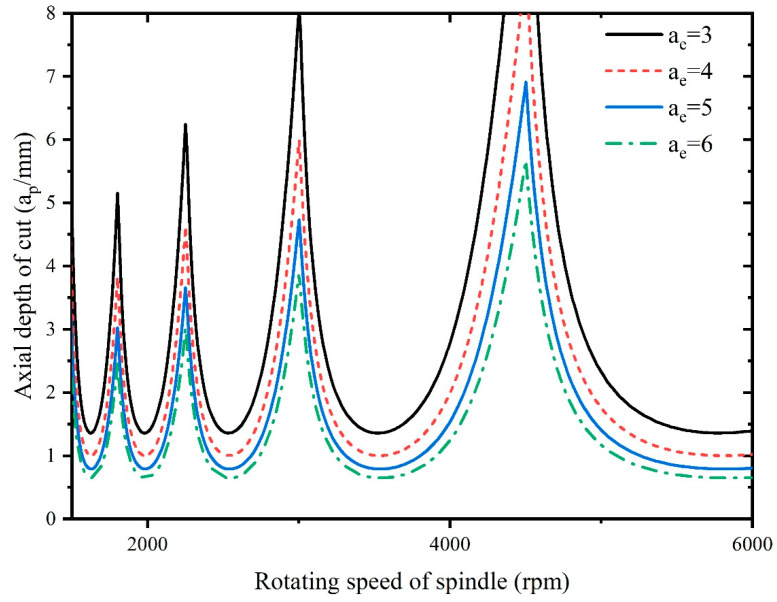
Influence of radial depth of cut on stability.

**Figure 18:**
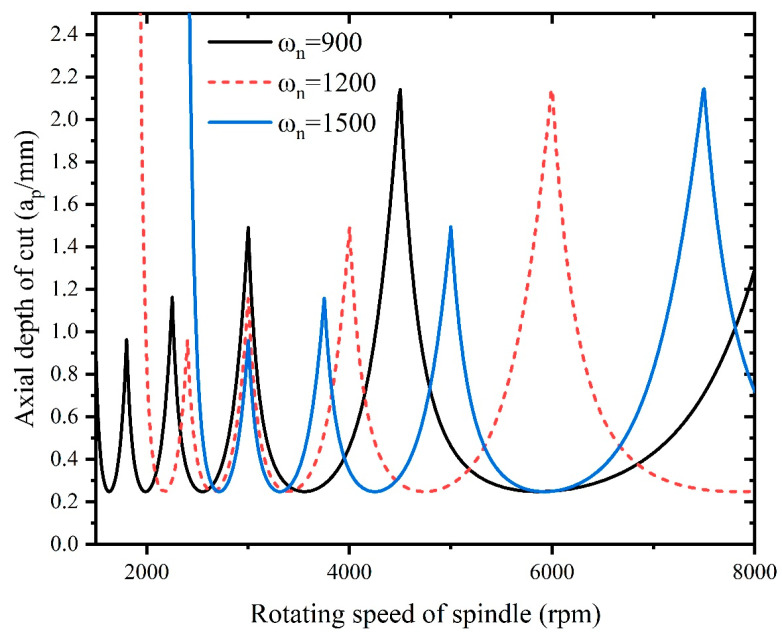
Influence of natural frequency on stability.

**Figure 19:**
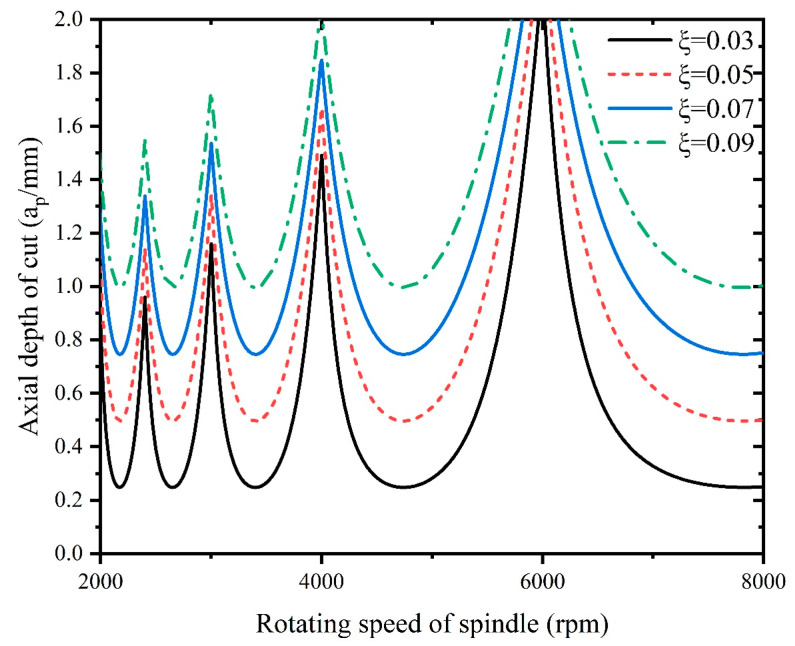
Influence of damping ratio on stability.

As it is necessary to select suitable processing parameters to avoid chattering during the milling process, therefore, there is a need to investigate the effect of the tool radius, radial depth of cut, natural frequency $\omega_{n}$, and damping ratio ζ on the cutting stability. The stability lobes are plotted for different tool radii, radial depths of cut, natural frequencies $\omega_{n}$, and damping ratios ζ, as shown in Figure 16, Figure 17, Figure 18 and Figure 19.

Figure 16 illustrates the influence of the tool radius on stability. As the tool radius increases, the system stability rises. This is because a larger tool radius provides greater stability by reducing the tendency for the tool to vibrate or chatter during machining. Inconel 718′s tough nature can amplify vibration problems, so using a larger tool radius helps dampen vibrations and leads to smoother cutting. Increasing the tool radius effectively decreases the depth of cut for a given engagement. This leads to a reduction in cutting forces as the material removal becomes more gradual, and the vibration can also be alleviated during the milling process.

Figure 17 demonstrates the influence of the radial depth of cut on stability, and it can be seen that the stability of the system decreases as the radial depth of cut increases, because a higher radial depth of cut leads to a larger chip thickness and engagement of the cutting tool with the workpiece. This generates higher cutting forces that can lead to tool deflection, chatter, and vibrations, reducing the stability of the machining process. With larger radial depths of cut, the likelihood of chattering and vibrations increases, and it can be especially problematic in milling hard-to-machine materials like Inconel 718. Additionally, larger radial depths of cut can lead to the formation of larger chips. Proper chip evacuation becomes challenging, and chip clogging can cause sudden force variations and affect system stability.

Figure 18 illustrates the influence of the natural frequency of the machine–tool system on stability, and it can be deduced that as the natural frequency increases, there is no significant change in the stability diagram, but the curve in the stability diagram is shifted to the right as the natural frequency increases. When the stability curve shifts to the right as the natural frequency increases, this suggests that the system’s inherent ability to resist vibrations or chatter improves. In other words, higher natural frequencies indicate that the system is less prone to self-excited vibrations that lead to chatter. This is because a higher natural frequency often implies that the system has better inherent damping characteristics. With increased damping, the system is more effective at absorbing and dissipating the energy generated during machining, leading to reduced vibrations and improved stability.

Figure 19 demonstrates the influence of the damping ratio on stability, and it can be concluded that an increasing damping ratio of the machine–tool system can improve the system stability. With increased damping, the system is more effective at absorbing and dissipating the energy generated during machining, leading to reduced vibrations and improved stability, because a higher damping ratio effectively increases the effective rigidity of the machine–tool system. This added rigidity counters the deflection caused by cutting forces and enhances the overall stability of the machining process.

## 5. Milling Experiment of Inconel 718 and Parameter Optimization

### 5.1. Methodology of Milling Experiment

To verify the correctness of the established milling force model and finite element analysis, a series of experiments including single factor experiments and orthogonal experiments are designed. After, the milling force measured from the experiments with that calculated from the theoretical model, as well as the milling forces obtained from the finite element analysis, are compared.

**Figure 20:**
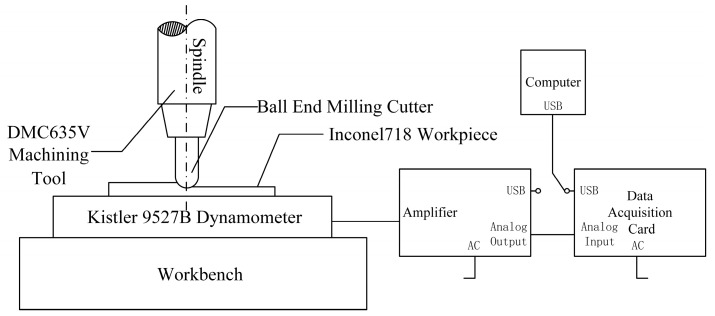
Schematic diagram of the connection of the experimental equipment.

**Figure 21:**
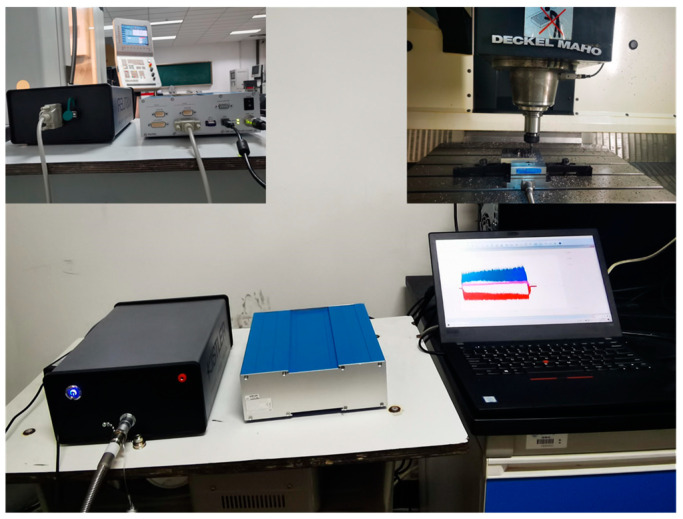
Connection of experimental equipment.

A DMC635V vertical machining center (developed by DMG in Germany), 1B240-0800-XA 1630 carbide ball-end milling cutter (developed by Sandvik in Germany), 9527B force gauge (developed by Kistler in Switzerland), and 5073A charge amplifier and acquisition card, as well as Dynoware computer analysis software (version 3.2.5.0), were selected to build the experimental platform. A schematic diagram of the connection of experimental equipment is shown in Figure 20, and a photo of the device’s connections is shown in Figure 21.

The single factor experiments and orthogonal experiments were designed as shown in Table 8 and Table 9.

### 5.2. Results of the Analysis of the Single Factor Experiment

**Figure 22:**
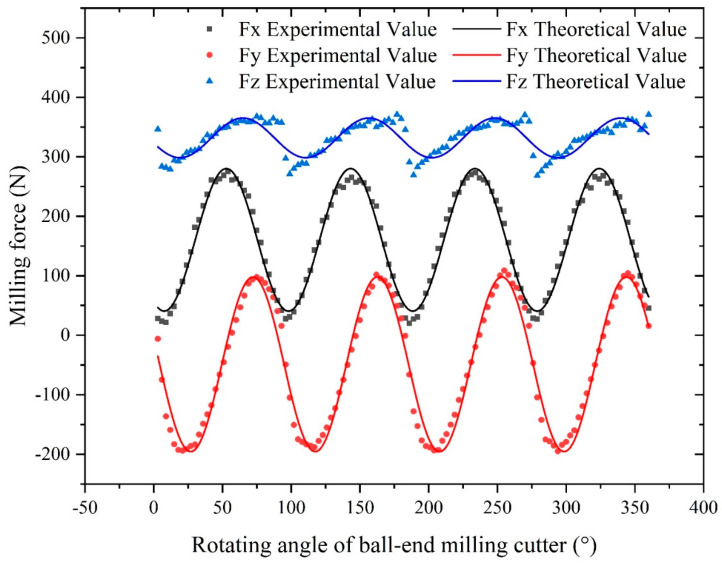
Comparison of the theoretical and experimental milling forces.

Based on the above-proposed milling force model of the ball-end milling cutter, the MATLAB program, which is illustrated in Appendix A, was used to calculate the theoretical milling force. The results of the theoretical analysis were compared to the experimentally measured results to verify the correctness of the proposed milling force model. The same manufacturing parameters were set for both the theoretical and experimental analyses, namely, the spindle speed was 1000 rpm, the axial depth of cut was 0.4 mm, and the feed per tooth was 0.04 mm/z. The results of the comparative analysis of the theoretical and experimental values of milling forces of the x-, y-, and z-axes are shown in Figure 22. Observing the milling force values for each axis, it is evident that both the theoretical and experimental results exhibit a similar change trend, validating the accuracy of the proposed theoretical model for calculating the ball-end milling cutter’s milling force. Although a slight degree of error is present in the figures, they remain within an acceptable range.

**Figure 23:**
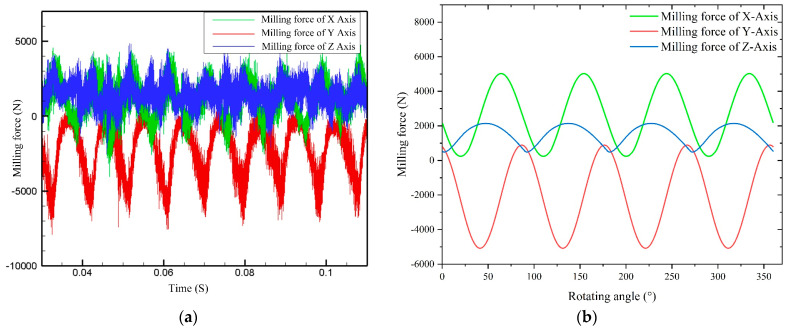
Comparison of the experimental and theoretical milling forces: (**a**) experimental milling force; (**b**) theoretical milling force.

The milling forces measured through milling experiment are presented in Figure 23a, and the milling forces calculated using the theoretical milling force model are presented in Figure 23b. The milling forces of each axis obtained from the theoretical model align closely with the experimental results, validating the accuracy and correctness of the proposed model.

**Figure 24:**
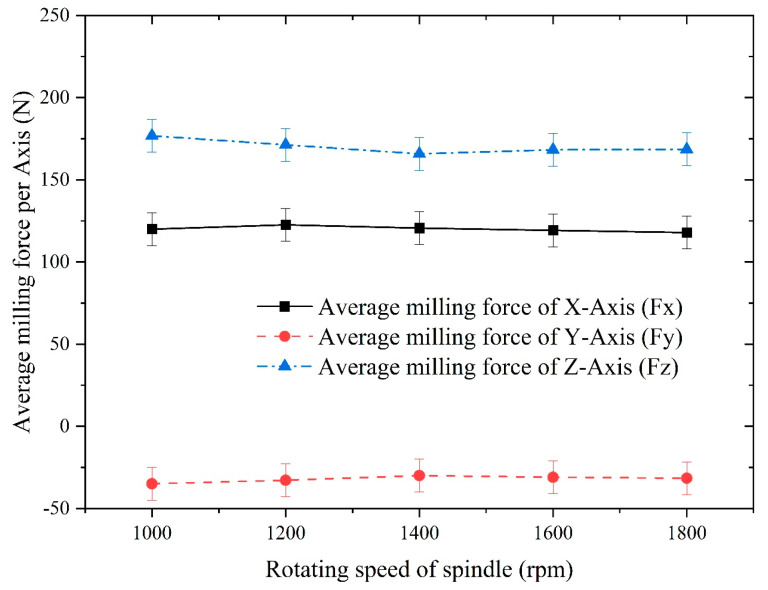
Influence of the spindle speed on the average milling force of each axis.

To investigate the influence of the spindle speed on the milling of Inconel 718, the parameter of spindle speed was varied at 1000 rpm, 1200 rpm, 1400 rpm, 1600 rpm, and 1800 rpm while keeping the other machining parameters unchanged. The axial depth of cut was set to 0.4 mm, and the feed per tooth was set to 0.02 mm/z. The experimental results of the milling force obtained under these conditions are presented in Figure 24. In Figure 24, the average milling force refers to the force calculated by selecting a stable range of cutting area presented in Dynoware software and making average the milling force in that area.

Figure 24 illustrates that the average milling forces for the x-, y-, and z-axes show minimal changes with an increasing spindle speed, which aligns with the conclusions drawn from the finite element analysis.

**Figure 25:**
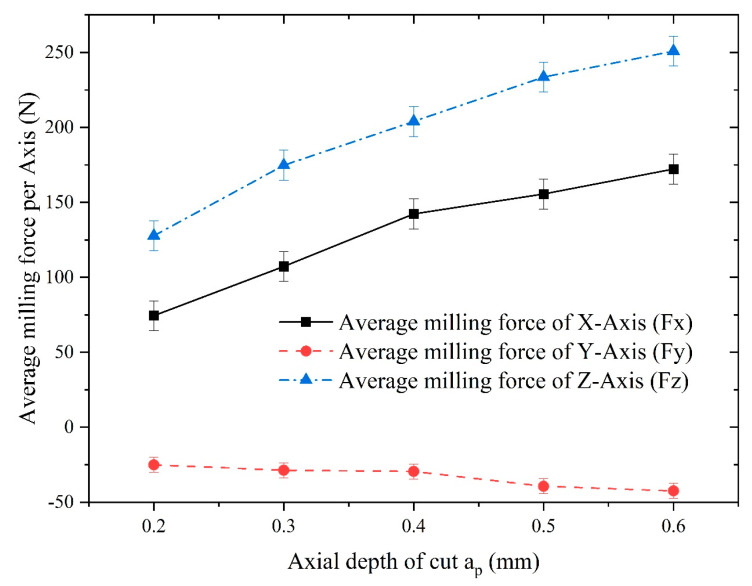
Influence of the axial depth of cut on the average milling force of each axis.

To unveil the influence of the axial depth of cut on the milling of Inconel 718, the parameter of the axial depth of cut was varied at 0.2 mm, 0.3 mm, 0.4 mm, 0.5 mm, and 0.6 mm while keeping the other machining parameters constant. The spindle speed was set to 1000 rpm, and the feed per tooth was set to 0.02 mm/z. The experimental results of the milling force obtained under these conditions are presented in Figure 25.

Figure 25 reveals that the average milling force for the x-, y-, and z-axes all demonstrated an increasing trend with the augmentation of the axial depth of cut. The z-axis exhibited the most significant increase, while the average milling forces in the x- and y-axes showed relatively smaller increments. These observations are consistent with the conclusions drawn from the finite element analysis.

**Figure 26:**
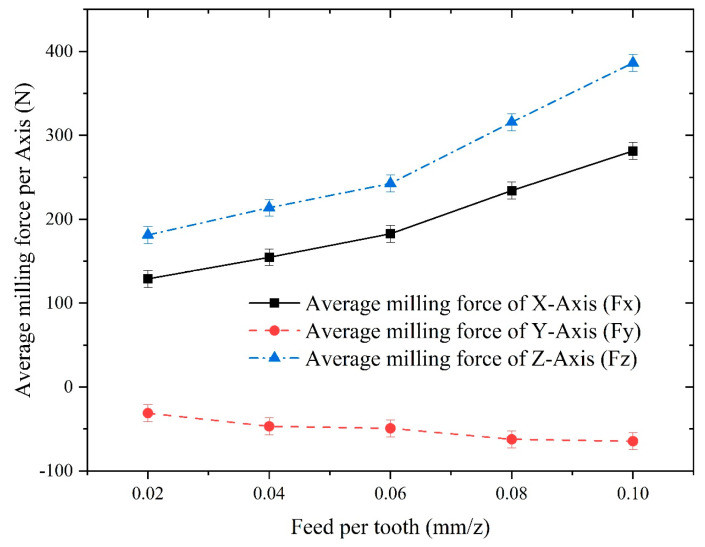
Influence of the feed per tooth on the average milling force of each axis.

To investigate the influence of the feed per tooth on the milling of Inconel 718, the parameter of feed per tooth was varied at 0.02 mm/z, 0.04 mm/z, 0.06 mm/z, 0.08 mm/z, and 0.10 mm/z while keeping the other machining parameters unchanged. The spindle speed was set to 1000 rpm, and the axial depth of cut was set to 0.4 mm. The experimental results of the milling force obtained under these conditions are presented in Figure 26.

It can be seen from Figure 26 that the average milling force for the x-, y-, and z-axes all reveal an increasing trend with the increase in feed per tooth, where the increase for the z-axis was the largest, and the increase for the x- and y- axes was relatively small, which is compatible with the conclusions drawn from the finite element analysis.

### 5.3. Results Analysis of the Orthogonal Experiments

The orthogonal experiments were analyzed based on the parameters shown in Table 9, and the orthogonal experimental results obtained are shown in Table 10.

An extreme variance analysis of the results shown in Table 10 lead to the following conclusions:

1. Axial depth of cut was the most influential factor on the average milling force for the x-axis, followed by feed per tooth and spindle speed.
2. Feed per tooth was the most influential factor on the average milling force for the y-axis, followed by axial depth of cut and spindle speed.
3. Axial depth of cut was the most influential factor on the average milling force for the z-axis, followed by the feed per tooth and spindle speed.

Considering that the effect of the feed per tooth and axial depth of cut on the average milling force in the previous analysis was large, while the effect of the spindle speed was relatively small, different parameters of the axial depth of cut and feed per tooth were selected for the verification experiments. Groups 1–4, as shown in Table 9, were selected, the theoretical and finite element analysis value of the average milling force of these groups were calculated in order to compare with the value of the experiments shown in Table 10, and the results are recorded in Table 11 and Table 12.

**Figure 27:**
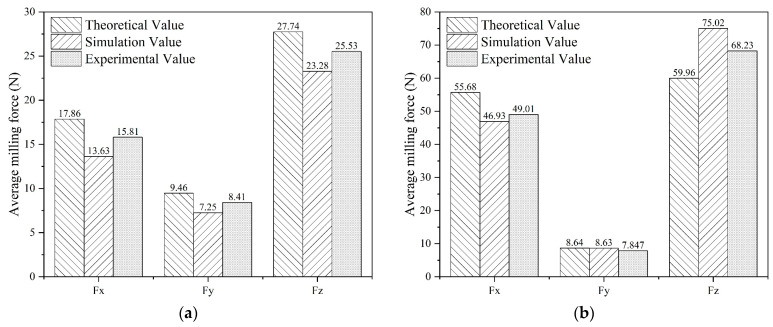

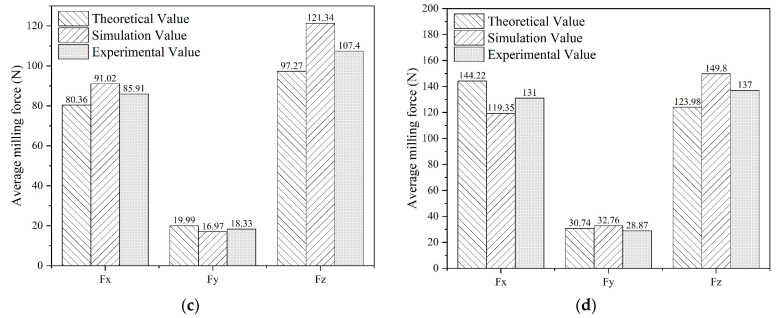
Comparison of the theoretical, FEA simulation, and experimental results of average milling force: (**a**) Group 1; (**b**) Group 2; (**c**) Group 3; (**d**) Group 4.

Figure 27 illustrates the comparison of the theoretical, finite element analysis simulation, and experimental results of the average milling force for the x-, y-, and z-axes in four different groups. Table 11 and Table 12, combined with Figure 27, reveal that the minimum relative error between the milling force calculated by the theoretical model and the experimental data is 6.48%, while the minimum relative error between the milling force obtained from finite element analysis and the experimental data is 4.24%. These results further demonstrate the reliability and validity of both the theoretical model and the finite element analysis.

### 5.4. Establishment of Optimization Objective Function

**Figure 28:**
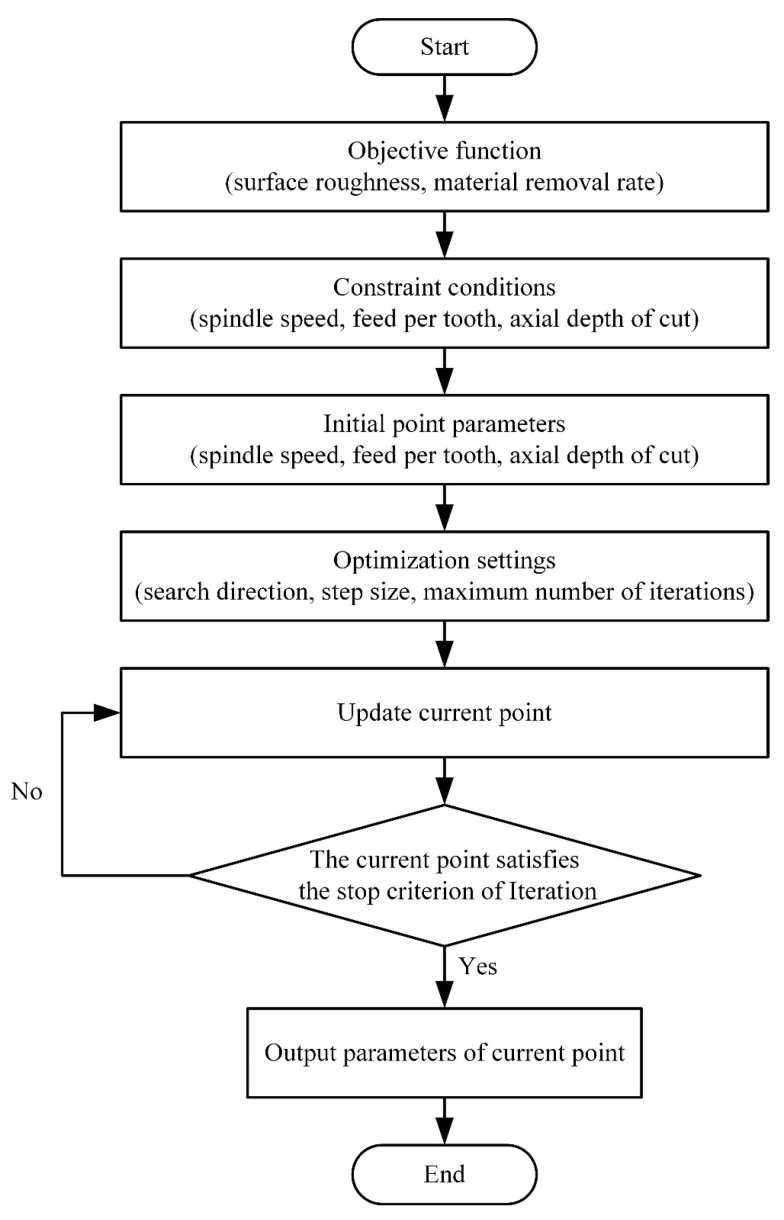
Flowchart of the procedure of optimization using the fmincon algorithm.

To enhance the machining efficiency and tool life expectancy of Inconel 718, optimizing the processing parameters becomes essential. In this study, surface roughness, material removal rate, and their combination were considered the optimization objectives. The fmincon algorithm, a nonlinear minimization method employing the interior point approach, was utilized to determine the optimal processing parameters. Known for its high accuracy in solving nonlinear optimization problems, the fmincon algorithm is well suited for this task. The optimization procedure employing the fmincon algorithm is depicted in Figure 28.

Surface roughness is an important evaluation index in the machining process of Inconel 718; therefore, a model with surface roughness as the optimization objective was developed, and the optimization objective function can be expressed as:

(24) $$R_{a}=C\cdot n^{c_{1}}\cdot f_{z}^{c_{2}}\cdot a_{p}^{c_{3}}$$

In Equation (24), the parameter C is influenced by both the geometry of the milling cutter and material of the workpiece. Parameters $c_{1}$, $c_{2}$, and $c_{3}$ are the influencing coefficients of the milling parameters.

Taking logarithms on both sides of Equation (24) yields:

(25) $$\ln R_{a}=\ln C+c_{1}\ln n+c_{2}\ln f_{z}+c_{3}\ln a_{p}$$

Taking $y=\ln R_{a}$, $c_{0}=\ln C$, $x_{1}=\ln n$, $x_{2}=\ln f_{z}$, and $x_{3}=\ln a_{p}$ and substituting these parameters into Equation (25) yields:

(26) $$y=c_{0}+c_{1}x_{1}+c_{2}x_{2}+c_{3}x_{3}$$

Based on this, multiple linear regression equations are built sequentially:

(27) $$\left\{\begin{array}{l} y_{1}=\beta_{0}+\beta_{1}x_{11}+\beta_{2}x_{12}+\beta_{3}x_{13}\\ y_{2}=\beta_{0}+\beta_{1}x_{21}+\beta_{2}x_{22}+\beta_{3}x_{23}\\ \;\;\vdots \\ y_{n}=\beta_{0}+\beta_{1}x_{n1}+\beta_{2}x_{n2}+\beta_{3}x_{n3} \end{array}\right.$$

Equation (27) is changed to a matrix representation:

(28) Y=Xβ+ε

The parameters in Equation (28) can be expressed as:

(29) $$Y=\left[\begin{array}{l} y_{1}\\ y_{2}\\ \;\vdots \\ y_{4} \end{array}\right],X=\left[\begin{array}{lllll} 1 & x_{11} & x_{12} & x_{13} & x_{14}\\ 1 & x_{21} & x_{22} & x_{23} & x_{24}\\ \;\vdots \\ 1 & x_{161} & x_{162} & x_{163} & x_{164} \end{array}\right],\beta =\left[\begin{array}{l} \beta_{0}\\ \beta_{1}\\ \;\vdots \\ \beta_{4} \end{array}\right],\varepsilon =\left[\begin{array}{l} \varepsilon_{1}\\ \varepsilon_{2}\\ \;\vdots \\ \varepsilon_{4} \end{array}\right]$$

Using the experimental results in Reference [52], the optimization objective Function (24) can be changed to:

(30) $$f_{1}\left(x\right)=R_{a}=\min\left(R_{a}\left(n,f_{z},a_{p}\right)\right)$$

In metal milling, improving productivity is critical, and the material removal rate is usually used to measure productivity. Therefore, a model with material removal rate as the optimization objective was developed:

(31) $$f_{2}\left(x\right)=\max\left(MRR\right)=\max\left(n\cdot f_{z}\cdot a_{p}\cdot N\right)$$

The parameter n in Equation (31) can be expressed as:

(32) $$n=\frac{1000v_{c}}{\pi D}$$

To ensure the machining stability, the processing parameters of Inconel 718 need to be selected within the stability region of the stability lobes, as shown in Figure 16, Figure 17, Figure 18 and Figure 19.

### 5.5. Optimization Results Analysis

**Figure 29:**
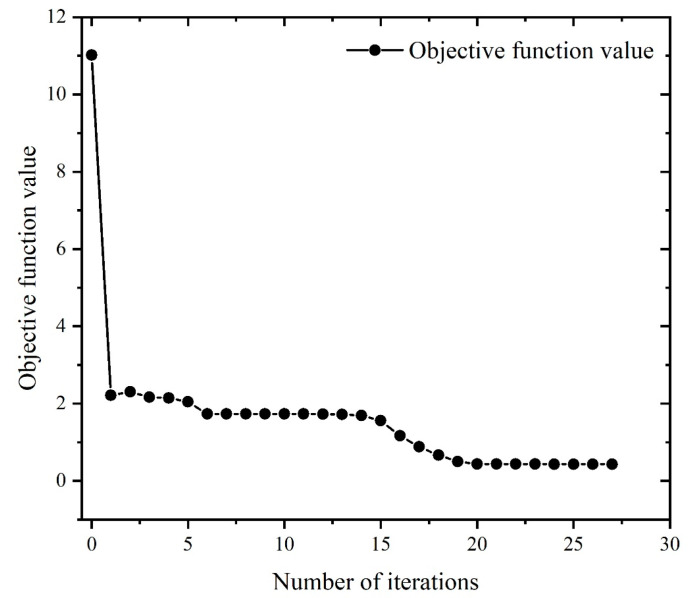
Iteration curve of the surface roughness.

When the minimum surface roughness is used as the optimization objective, it can be observed from Figure 29 that the solution of the optimization objective function gradually converges as the number of iterations increases. According to the optimal milling parameters recorded in Table 13, it can be concluded that the surface roughness reached the optimal value at the 19th generation, and the optimal surface roughness is 0.43 μm.

**Figure 30:**
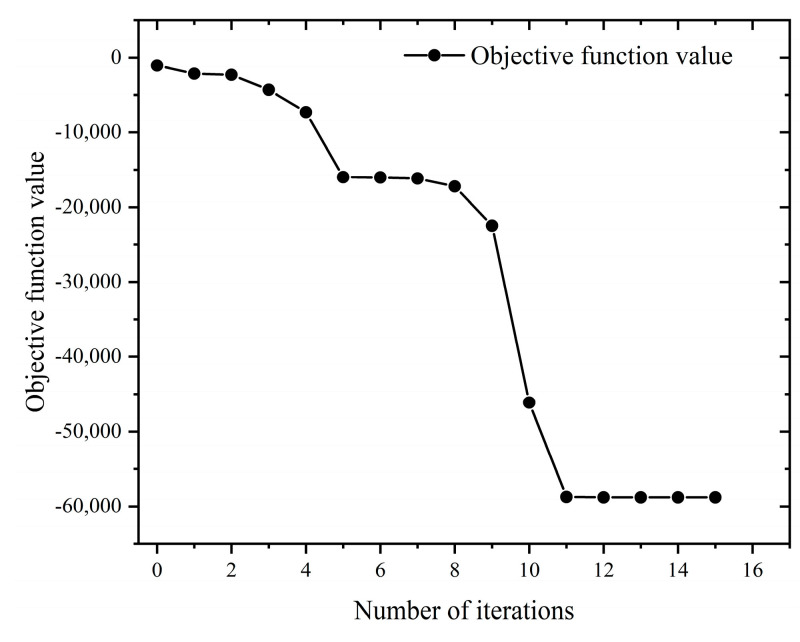
Iterative curve of material removal rate.

When the maximum material removal rate was used as the optimization objective, it can be seen from Figure 30 that the solution of the optimization objective function gradually converged as the number of iterations increased. According to the optimal milling parameters recorded in Table 14, it can be concluded that the material removal rate reached the optimal value at the 11th generation, and the optimal material removal rate is 58,788.32 (mm^3^/min).

If the maximum material removal rate and minimum surface roughness are taken as the research objective of the multi-objective optimization, the mathematical model of the multi-objective optimization is as follow:

(33) $$\begin{array}{l} \min f(x)=\min(\omega_{1}f_{1}(x)+\omega_{2}f_{2}(x))\\ s.t.\left\{\begin{array}{l} n_{\min}\leq n\leq n_{\max}\\ f_{z}\leq \frac{\pi Dv_{f\max}}{1000vz}\\ a_{p\min}\leq a_{p}\leq a_{p\max} \end{array}\right. \end{array}$$

In Equation (33), the parameters $\omega_{1}$ and $\omega_{2}$ are weighting values of the machining efficiency and surface roughness, respectively.

**Figure 31:**
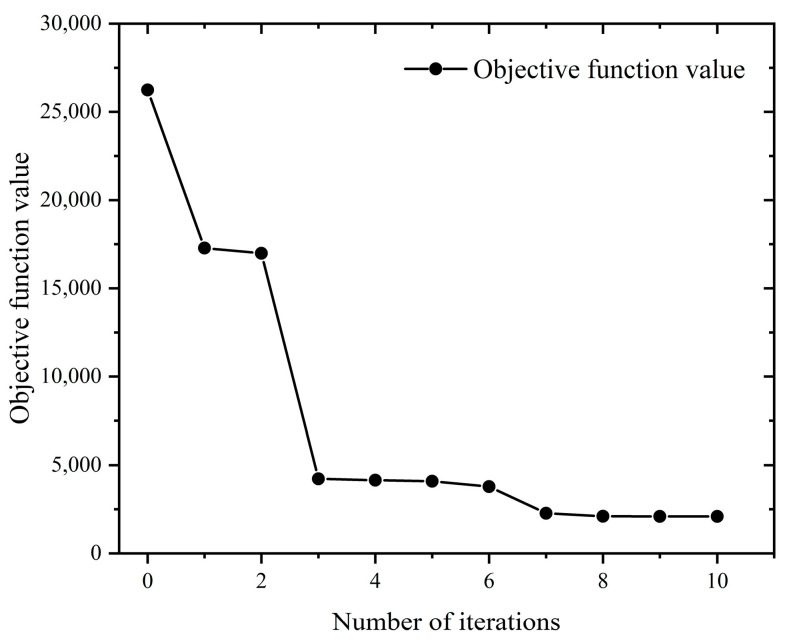
Multi-objective optimization iteration curve.

It can be seen from Figure 31 that the solution of the optimization objective function gradually converges as the number of iterations increases. According to the optimal milling parameters recorded in Table 15, it can be concluded that the optimal material removal rate reached 4199.2 mm^3^/min, while the optimal surface roughness reached 3.5 μm.

## 6. Conclusions

This study addresses a critical gap in the realm of metal cutting, specifically focusing on the machining of nickel-based superalloy Inconel 718. While previous research has made strides in modeling milling forces and stability analysis, a distinct gap persists in the understanding of milling mechanisms and chattering stability of Inconel 718. This work introduced a tailored milling force model for ball-end milling cutters, offering insights into machining forces for Inconel 718. By coupling finite element analysis with material failure criteria, the intricate interplay between processing parameters and material behavior during Inconel 718 machining is elucidated. A noteworthy contribution of this study lies in its meticulous examination of chattering stability during the machining process. The establishment of stability lobe diagrams through dynamic equations enhances the selection of stable cutting parameters, bolstering machining outcomes for challenging materials like Inconel 718. Furthermore, this study pioneered a multi-objective optimization approach, concurrently considering conflicting objectives such as surface roughness and material removal rate. By employing this algorithm, optimal machining parameters were derived, signifying a path towards improved machining efficiency and quality for Inconel 718.

The main conclusions are as follow:

1. The finite element analysis revealed notable temperature increases in the machining zone with rising spindle speed, radial and axial depths of cut, and feed per tooth. Tool stress, influenced mainly by feed per tooth and radial and axial depths of cut, exhibited significant changes. While spindle speed moderately affected milling force for each axis, the feed per tooth and radial depth of cut substantially increased forces in the x-, y-, and x-axes.
2. Chattering stability was investigated, with tool radius enhancement aiding stability, while radial depth of cut undermined it. An improved damping ratio enhances stability, while the system’s inherent ability to resist vibrations or chatter improves with the increase in natural frequency.
3. Validation against experiments and calculated results from the milling force model and finite element analysis exhibited minor deviations.
4. The machining parameters optimized using the fmincon algorithm: for minimum surface roughness, spindle speed of 3999.63 rpm, feed rate of 80.01 mm/min, axial depth of cut of 0.25 mm, and yield surface roughness of 0.43 μm; for maximum material removal rate, spindle speed of 4000 rpm, feed speed of 700 mm/min, axial depth of cut of 2.54 mm, and yield of 58,788.32 mm³/min. Joint optimization yields, spindle speed of 3199.2 rpm, feed speed of 80 mm/min, and axial depth of cut of 0.25 mm with a surface roughness of 3.5 μm and material removal rate of 4199.2 mm³/min.

However, despite these advancements, certain aspects warrant further exploration. While this study provides a solid foundation, more extensive experimental validation across a range of conditions would enhance the reliability of the proposed models and findings. Additionally, the application of advanced machine learning techniques to predict machining behavior could further enhance the accuracy and efficiency of machining processes for Inconel 718.

## Figures and Tables

**Table 1:** Data from the recognition experiments of the milling force coefficients.

Experiment No.	$f_{t}$ (mm/z)	Feed Speed (mm/min)	$\bar{F_{x}}\left(N\right)$	$\bar{F_{y}}\left(N\right)$	$\bar{F_{z}}\left(N\right)$
1	0.02	80	76.88	−59.25	11.97
2	0.04	160	174.5	−210.7	44.67
3	0.06	240	251.7	−378.8	133.1
4	0.08	320	314.1	−572.0	265.2
5	0.10	400	353.4	−720.6	430.1
6	0.12	480	421.6	−830.1	552.3

**Table 2:** Coefficients of the milling force.

Shearing Force Coefficient (N/mm^2^)	Value	Edge Force Coefficient (N/mm^2^)	Value
$K_{tc}$	−15,934.72	$K_{te}$	149.15
$K_{rc}$	−6636.28	$K_{re}$	−51.95
$K_{ac}$	8949.06	$K_{ae}$	−159.45

**Table 3:** Processing parameters used in the finite element analysis.

Rotating Speed of Spindle *n* (rpm)	$\mathrm{Feed}\;\mathrm{per}\;\mathrm{Tooth}\;f_{t}$ (mm/z)	$\mathrm{Radial}\;\mathrm{Depth}\;\mathrm{of}\;\mathrm{Cut}\;a_{e}$(mm)	$\mathrm{Axial}\;\mathrm{Depth}\;\mathrm{of}\;\mathrm{Cut}\;a_{p}$(mm)
1000	0.2	2	2
1300	0.3	3	3
1600	0.4	4	4
-	0.5	5	5

**Table 4:** Strain enhancement parameters of Inconel 718.

$\mathrm{Initial}\;\mathrm{Yield}\;\mathrm{Stress}\;\sigma_{0}$	$\mathrm{Reference}\;\mathrm{Plastic}\;\mathrm{Strain}\;\varepsilon_{0}^{p}$	Cut-Off Strain $\varepsilon_{cut}^{p}$	Strain Enhancement Index n
3.6 MPa	$5.288\times 10^{-3}$	0.3	9.55

**Table 5:** Thermal softening parameters of Inconel 718.

$c_{0}$	$c_{1}$	$c_{2}$	$c_{3}$	$c_{4}$	$c_{5}$	Ambient Temperature T	Linear Cut-Off Temperature $T_{cut}$	$\mathrm{Melting}\;\mathrm{Temperature}\;T_{melt}$
0.998	$2.46\times 10^{-4}$	$-3.74\times 10^{-6}$	$1.51\times 10^{-8}$	$-2.35\times 10^{-11}$	$1.10\times 10^{-14}$	20 °C	1050 °C	1298 °C

**Table 6:** Strain rate parameters of Inconel 718.

$\mathrm{Low}\;\mathrm{Strain}\;\mathrm{Rate}\;\mathrm{Sensitivity}\;\mathrm{Factor}\;m_{1}$	$\mathrm{High}\;\mathrm{Strain}\;\mathrm{Rate}\;\mathrm{Sensitivity}\;\mathrm{Factor}\;m_{2}$	$\mathrm{Reference}\;\mathrm{Plastic}\;\mathrm{Strain}\;\mathrm{Rate}\;{\dot{\varepsilon }}_{0}$	$\mathrm{Strain}\;\mathrm{Rate}\;\mathrm{Critical}\;\mathrm{Value}\;{\dot{\varepsilon }}_{t}$
25.5	25.5	1 s^−1^	1 × 10^7^ s^−1^

**Table 7:** Results of the modal experiments.

Modal Order	Natural Frequency (Hz)	Damping Ratio (*%*)
First order of x-direction	935.06	0.049
Second order of x-direction	1235.96	0.036
Third order of x-direction	1567.99	0.093
First order of y-direction	911.87	0.045
Second order of y-direction	1233.52	0.059
Third order of y-direction	1539.92	0.050

**Table 8:** Parameters of the single factor experiments.

Experiment No.	Rotating Speed of Spindle N (r/min)	Feed per Tooth $f_{z}$ (mm/z)	Axial Depth of Cut $a_{p}$ (mm)
1	1000/1200/1400/1600/1800	0.02	0.4
2	1000	0.02/0.04/0.06/0.08/0.10	0.4
3	1000	0.02	0.2/0.3/0.4/0.5/0.6

**Table 9:** Parameters of the orthogonal experiments.

Experiment No.	Rotating Speed of Spindle N (rpm)	Feed per Tooth $f_{z}$ (mm/z)	Axial Depth of Cut $a_{p}$ (mm)
1	800	0.015	0.1
2	800	0.03	0.2
3	800	0.045	0.3
4	800	0.06	0.4
5	1000	0.015	0.2
6	1000	0.03	0.1
7	1000	0.045	0.4
8	1000	0.06	0.3
9	1200	0.015	0.3
10	1200	0.03	0.4
11	1200	0.045	0.1
12	1200	0.06	0.2
13	1400	0.015	0.4
14	1400	0.03	0.3
15	1400	0.045	0.2
16	1400	0.06	0.1

**Table 10:** Result of the orthogonal experiments.

Experiment No.	Parameters of Experiment			Result of Experiment (N)			
	Rotating Speed of Spindle n (rpm)	Feed per Tooth $f_{z}$ (mm/z)	Axial Depth of Cut $a_{p}$ (mm)	$\bar{F_{x}}$	$\bar{F_{y}}$	$\bar{F_{z}}$	$\bar{F}$
1	800	0.015	0.1	15.81	8.410	25.53	31.18
2	800	0.03	0.2	49.01	−7.847	68.23	84.37
3	800	0.045	0.3	85.91	−18.33	107.4	138.75
4	800	0.06	0.4	131.0	−28.87	137.0	191.74
5	1000	0.015	0.2	57.65	−15.96	87.60	106.08
6	1000	0.03	0.1	39.64	−8.765	65.59	77.14
7	1000	0.045	0.4	58.2	42.99	70.62	101.11
8	1000	0.06	0.3	118.4	−13.09	148.7	190.53
9	1200	0.015	0.3	79.75	−16.09	117.9	143.25
10	1200	0.03	0.4	121.9	−24.2	157.3	200.47
11	1200	0.045	0.1	38.06	−9.168	66.27	76.97
12	1200	0.06	0.2	53.36	35.24	69.54	94.47
13	1400	0.015	0.4	86.23	2.896	120.7	148.37
14	1400	0.03	0.3	54.12	40.37	82.35	106.49
15	1400	0.045	0.2	87.04	−23.66	134.8	162.19
16	1400	0.06	0.1	66.65	−12.69	101.1	121.76

**Table 11:** Comparison between the theoretical and experimental results of the milling force.

Experiment No.	Milling Force	Theoretical Result (N)	Experimental Result (N)	Relative Error (%)
1	$F_{x}$	17.86	15.81	12.97
	$F_{y}$	9.46	8.41	12.49
	$F_{z}$	27.74	25.53	8.66
2	$F_{x}$	55.68	49.01	13.61
	$F_{y}$	8.64	7.847	10.11
	$F_{z}$	59.96	68.23	12.12
3	$F_{x}$	80.36	85.91	6.46
	$F_{y}$	19.99	18.33	9.06
	$F_{z}$	97.27	107.4	9.43
4	$F_{x}$	144.22	131	10.09
	$F_{y}$	30.74	28.87	6.48
	$F_{z}$	123.98	137	9.50

**Table 12:** Comparison between the finite element analysis and experimental results of the milling force.

Experiment No.	Milling Force	FEA Result (N)	Experimental Result (N)	Relative Error (%)
1	$F_{x}$	13.63	15.81	13.80
	$F_{y}$	7.25	8.41	13.79
	$F_{z}$	23.28	25.53	8.81
2	$F_{x}$	46.93	49.01	4.24
	$F_{y}$	8.63	7.847	9.98
	$F_{z}$	75.02	68.23	9.95
3	$F_{x}$	91.02	85.91	5.95
	$F_{y}$	16.97	18.33	7.42
	$F_{z}$	121.34	107.4	12.98
4	$F_{x}$	119.35	131	8.89
	$F_{y}$	32.76	28.87	13.47
	$F_{z}$	149.8	137	9.34

**Table 13:** Results of the optimization of the surface roughness.

	Spindle Speed (rpm)	Feed Speed (mm/min)	Axial Depth of Cut (mm)	Surface Roughness R (μm)
Initial Value	1000	100	1.2	11
Optimized Value	3999.63	80.01	0.25	0.43

**Table 14:** Results of the optimization of the material removal rate.

	Spindle Speed (rpm)	Feed Speed (mm/min)	Axial Depth of Cut (mm)	Material Removal Rate (mm^3^/min)
Initial Value	1000	100	1.2	1049.79
Optimized Value	4000	700	2.54	58,788.32

**Table 15:** Results of the multi-objective optimization.

	Spindle Speed (rpm)	Feed per Tooth (mm/z)	Axial Depth of Cut (mm)	Material Removal Rate (mm^3^/min)	Surface Roughness (μm)
Initial Value	1000	100	1.2	10,498	11
Optimized Value	3199.2	80	0.25	4199.2	3.5

## Data Availability

Not applicable.

## Nomenclature

The nomenclature used in this article is shown below:

$dF_{t,j}\left(\phi_{j}\left(z\right)\right)$	The differential tangential cutting force (N)
$dF_{r,j}\left(\phi_{j}\left(z\right)\right)$	The differential radial cutting force (N)
$dF_{a,j}\left(\phi_{j}\left(z\right)\right)$	The differential axial cutting force (N)
$K_{tc}$	Tangential shearing force coefficient (N/mm^2^)
$K_{rc}$	Radial shearing force coefficient (N/mm^2^)
$K_{ac}$	Axial shearing force coefficient (N/mm^2^)
$K_{te}$	Tangential edge force coefficient (N/mm)
$K_{re}$	Radial edge force coefficient (N/mm)
$K_{ae}$	Axial edge force coefficient (N/mm)
$h_{j}\left(\phi_{j}\left(z\right)\right)$	The instantaneous chip thickness at immersion angle (mm)
$dS\left(\phi_{j}\left(z\right)\right)$	The instantaneous edge length of the cutting segment (mm)
db	The instantaneous chip width (mm)
$F_{f}\left(\phi \right)$	The cutting force in feed direction (N)
$F_{n}\left(\phi \right)$	The cutting force in normal direction (N)
$F_{z}\left(\phi \right)$	The cutting force in axial direction (N)
$\bar{F_{f}}$	The average cutting force in the feed direction (N)
$\bar{F_{n}}$	The average cutting force in the normal direction (N)
$\bar{F_{z}}$	The average cutting force in the axial direction (N)
$\bar{F_{fc}}$, $\bar{F_{fe}}$	The components of linear model force in the feed direction (N)
$\bar{F_{nc}}$, $\bar{F_{ne}}$	The components of linear model force in the normal direction (N)
$\bar{F_{zc}}$, $\bar{F_{ze}}$	The components of linear model force in the axial direction (N)
$\sigma \left(\varepsilon^{p},\dot{\varepsilon },T\right)$	Workpiece material flow stress
$g\left(\varepsilon^{p}\right)$	Strain enhancement function
$\Gamma (\dot{\varepsilon })$	Strain rate effect function
Θ(T)	Thermal softening function
$\dot{\varepsilon }$	Strain rate
T	Temperature
$\sigma_{0}$	Initial yield stress
$\varepsilon^{p}$	Plastic strain
$\varepsilon_{0}^{p}$	Reference plastic strain
$\varepsilon_{cut}^{p}$	Cut-off strain value
n	Contingency strengthening index
$\Delta \varepsilon_{i}^{p}$	Instantaneous strain increment
$\varepsilon_{f_{i}}^{p}$	Material failure strain
$m_{x}$, $m_{y}$	Mass of machine–tool system in the x- and y-directions
$c_{x}$, $c_{y}$	Damping of machine–tool system in the x- and y-directions
$k_{x}$, $k_{y}$	Stiffness of machine–tool system in the x- and y-directions
$F_{xj}$, $F_{yj}$	Cutting force components acting on the tooth in the x- and y-directions
$v_{j,0}$	Dynamic displacement of the cutter in the previous cycle
$v_{j}$	Dynamic displacement of the cutter in the current cycle
$g\left(\phi_{j}\right)$	Unit step function
$\Phi_{xx}\left(i\omega \right)$	Direct transfer function in the x-direction
$\Phi_{yy}\left(i\omega \right)$	Direct transfer function in the y-direction
$\Phi_{xy}\left(i\omega \right)$	Cross-transfer function in the x-direction
$\Phi_{yx}\left(i\omega \right)$	Cross-transfer function in the y-direction
$v_{c}$	Speed of milling process
n	Rotating speed of spindle
$f_{z}$	Feed per tooth
$a_{e}$	Radial depth of cut
$a_{p}$	Axial depth of cut

## Appendix A

The MATLAB program used to solve milling force model of the ball-end milling cutter is shown below:

clear;

clc;

R = 4;

%ap = 0.2;

zt1 = −R:0.01:0;

phi_2 = linspace(0,2*pi,100);

n11 = 1;

%Cutter Parameters

beta = 45/180*pi;D = 10;Nf = 2;R = D/2;

%Cutting Parameters

ap = 0.2;fz = 0.1;n = 1200;

%Coefficient of Milling Force

Kts = 3304.12;

Krs = 2148.90;

Kas = 725.07;

Kre = 67.58;

Kte = 5.28;

Kae = −7.16;

%Differential Elements

dphi = 0.01;dz = 0.01;

K = 700;

dphi = 4*pi/K;

zt1 = −R:dz:0;

phi_p = 2*pi/Nf;wr = 2*pi*n/60;

for m = 1:K%Discretization of rotating angle

phi(m) = m*dphi;

Fx(m) = 0;Fy(m) = 0;Fz(m) = 0;

dFx = 0;dFy = 0;dFz = 0;

for k = 1:2%Number of teeth cycle

phi_11 = phi(m) + (k − 1)*phi_p;%Rotation angle considering the number of teeth

for i = 2:length(zt1)

z = zt1(i);

zt = z;

phi_3 = phi_11 − (z + R)*tan(beta)/R;

ks = acos(-zt/R);

if mod(phi_3,2*pi) > =0& mod(phi_3,2*pi) < =pi

dS = sqrt(1 + z^2/(R^2 − z^2) + tan(beta)^2/R^2*(R^2 − z^2))*dz;

db = R/(R^2 − z^2)*dz;

%Calculation of cutting thickness

tn = fz*sin(phi_3)*sin(ks);

%End of calculation of cutting thickness

k1 = acos(−z/R);

dFr = Kre*dS + Krs*tn*db;

dFt = Kte*dS + Kts*tn*db;

dFa = Kae*dS + Kas*tn*db;

dFx = −cos(phi_3)*dFt-sin(k1)*sin(phi_3)*dFr-cos(k1)*sin(phi_3)*dFa;

dFy = sin(phi_3)*dFt-sin(k1)*cos(phi_3)*dFr-cos(k1)*cos(phi_3)*dFa;

dFz = cos(k1)*dFr-sin(k1)*dFa;

else

dFx = 0;

dFy = 0;

dFz = 0;

end

Fx(m) = Fx(m) + dFx;

Fy(m) = Fy(m) + dFy;

Fz(m) = Fz(m) + dFz;

end

end

end

phi_x = phi/pi*180/2;

plot(phi_x,Fx,‘r’,‘MarkerSize’,3,‘LineWidth’,2);

hold on;

plot(phi_x,Fy,‘b’,‘MarkerSize’,3,‘LineWidth’,2);

hold on;

plot(phi_x,Fz,‘k’,‘MarkerSize’,3,‘LineWidth’,2);

hold on;

set(gca,‘linewidth’,0.5,‘fontsize’,15,‘fontname’,‘Times’);
